# Serovar-specific genomic features of *Leptospira interrogans* Hardjo: implications for host adaptation

**DOI:** 10.3389/fmolb.2025.1648097

**Published:** 2025-09-10

**Authors:** Klaudia Dubniewicz, Laura Pardyak, Artur Gurgul, Igor Jasielczuk, Tomasz Szmatoła, Zbigniew J. Arent

**Affiliations:** ^1^ Department of Infectious Diseases and Public Health, Faculty of Veterinary Medicine, University of Agriculture, Krakow, Poland; ^2^ Department of Basic Sciences, Faculty of Veterinary Medicine, University of Agriculture, Krakow, Poland

**Keywords:** *Leptospira interrogans*, Hardjo, Comparative genomics, whole genome sequencing, Europe

## Abstract

**Introduction:**

Leptospirosis, caused by *Leptospira* spp.*,* is one of the most widespread zoonoses worldwide. It affects both domestic and wild animals, with ruminants serving as a primary reservoir for serovar Hardjo. This serovar causes long-term colonisation of the kidney and genital tract. Hardjo strains are taxonomically assigned to two *Leptospira* species: *Leptospira interrogans* and *Leptospira borgpetersenii*. However, the molecular basis of *L. interrogans serovar* Hardjo adaptation remains poorly understood. Comparative genomic analysis of *L. interrogans* strains classified as the Hardjo serovar and other non-Hardjo serovars of the same species may help identify genetic determinants associated with host adaptation and species-specific cellular immune responses. Unfortunately, these pathogens are highly fastidious, and only a limited number of whole genomes have been sequenced to date.

**Materials and Methods:**

Four *L. interrogans* serovar Hardjo European isolates were sequenced. Using these new sequences alongside publicly available genomes of *L. interrogans* strains classified as Hardjo and non-Hardjo serovars, we performed comparative genomic analyses.

**Results:**

Hardjo strains formed a distinct phylogenetic clade and harboured unique variants, including an intact *cas3* gene and a modified *thiM* start codon. We identified 88 Hardjo-specific orthologues, some located in putative genomic islands outside *rfb* locus. Several encoded proteins related to mobile elements, toxin–antitoxin systems or signal transduction. Enhanced biofilm formation in Hardjo strains supports a host-adapted phenotype.

**Conclusion:**

This study expands the genomic dataset for *L. interrogans* serovar Hardjo and provides novel insights into its genetic distinctiveness, suggesting potential factors that may facilitate colonisation and persistence in ruminant hosts.

## 1 Introduction

Leptospirosis is a globally distributed zoonotic disease caused by bacteria of the genus *Leptospira*. Infections occur in a wide range of domestic and wild animals, making it one of the most prevalent zoonoses worldwide (Costa et al., 2015). Pathogenic *Leptospira* species colonise the kidneys and genital tracts of carrier animals and are shed in urine (Monahan et al., 2009) and reproductive fluids (Dhaliwal et al., 1996; Ellis et al., 1986; Loureiro et al., 2017; Masri et al., 1997), thereby facilitating transmission to new hosts. Although these bacteria can infect many different animal species, each pathogenic serovar is typically maintained within specific animal reservoirs (Ellis, 2015).

Infection in cattle was recognised in 1935 (Mikhin and Azinov, 1935). Bovine infections may result from incidental exposure to serovars (sv.) such as Icterohaemorrhagiae, Pomona, or Grippotyphosa, which are maintained in other animal species (Arent et al., 2017). However, in the 1960s, cattle were recognised as reservoir hosts for the *Leptospira* serovar Hardjo (Roth and Galton, 1960). This serovar is adapted to cattle and sheep and is capable of long-term colonisation of both the kidneys and reproductive organs. Loureiro and Lilenbaum (Loureiro and Lilenbaum, 2020) proposed that chronic genital leptospirosis in cattle constitutes a distinct syndrome, separate from systemic infection, and thus requires specialised diagnostic and therapeutic strategies.

The introduction of genetic typing methods led to the identification of two genetically distinct but serologically indistinguishable types within serovar Hardjo: Hardjo type Bovis and Hardjo type Prajitno (Thiermann et al., 1986). Based on genomic classification and species definition criteria (Yasuda et al., 1987), these types were assigned to two different species - *L. borgpetersenii* (Hardjo type Bovis, HB) and *L. interrogans* (Hardjo type Prajitno, HP). *L. interrogans* serovar Hardjo was first identified in Scotland and Northern Ireland and has since been isolated from cattle and sheep across Europe and South America (Ellis, 2015; Salgado et al., 2015). Although early isolates primarily belonged to this species, *Leptospira borgpetersenii* serovar Hardjo is the most prevalent representative of this serovar maintained by cattle and sheep with nearly global distribution (Ellis, 2015).

The ecological adaptability of *Leptospira* has been linked to the genetic diversity encoded in its relatively large genome - averaging 4.26 Mb, with sizes ranging from 3.89 Mb in *L. borgpetersenii* to 4.71 Mb in *L. interrogans* (Fouts et al., 2016; Philip et al., 2021). The reduced genome of *L. borgpetersenii* is associated with increased host dependence and restricted environmental survival, significantly influencing transmission dynamics (Bulach et al., 2006). In contrast, *L. interrogans* retains a larger genome, enabling the acquisition of genes involved in immune evasion and host adaptation (Giraud-Gatineau et al., 2024; Xu et al., 2016).

Despite its clinical and epidemiological relevance, the host-pathogen relationship between cattle and *Leptospira* serovar Hardjo remains poorly understood, limiting progress in vaccine development and effective disease control. Unlike other serovars - such as Icterohaemorrhagiae, Pomona, Canicola, and Grippotyphosa, that primarily induce humoral immunity through antibodies against lipopolysaccharides (LPS), protection against Hardjo appears to rely on strong cell-mediated immune responses (Bolin et al., 1989; Naiman et al., 2001). Although there is compelling evidence for a protective protein antigen (Kunjantarachot et al., 2014; Wunder et al., 2021), no specific antigen has yet been identified (Murray et al., 2013), further underscoring the need to understand serovar-specific immune mechanisms. At the molecular level, the mechanisms of host adaptation by *Leptospira* sv. Hardjo remain largely uncharacterised. Comparative genomic analyses, particularly between *L. interrogans* sv. Hardjo and other serovars, could help identify genes involved in host adaptation and immune modulation in cattle and sheep. However, research in this area has been constrained by the fastidious nature of these organisms and the limited availability of fully sequenced genomes, especially for serovar Hardjo. Although previous genomic studies have examined *L. interrogans* sv. Hardjo (Cosate et al., 2015; Llanes et al., 2016), they lacked European strains and broader representation of Hardjo isolates, leaving important questions unanswered.

This study presents a comparative analysis of *L. interrogans* serovar Hardjo strains based on whole-genome sequencing. The primary objectives were to sequence and assemble genomes from selected Hardjo isolates, assess their phylogenetic relationships, and identify genes and gene products potentially involved in the specific infection process. Here, we report the genome sequences of four European *L. interrogans* serovar Hardjo isolates. Our central hypothesis is that comparative genomic analysis of *L. interrogans* serovar Hardjo with other *L. interrogans* serovars sharing a similar genomic background could reveal genetic determinants specific to the Hardjo serovar, which may underlie its unique host associations and pathogenic traits. We hope that this research will serve as a foundation for further investigations into the molecular mechanisms underlying Hardjo serovar determination, which may be conserved across both *L. interrogans* and *L. borgpetersenii* species.

## 2 Materials and methods

### 2.1 Sample characterisation

The study was based on four *L. interrogans* serovar Hardjo strains: KR40, KR84, KR85, and N116 – each isolated from different host and region in Europe. Strain KR40 was obtained from a horse in Italy in 1997, KR84 from a wallaby in the United Kingdom in 1983, KR85 from a dog in the United Kingdom in 1982, and N116 from a cow in Belgium in 2016. All *Leptospira* isolates were originally classified on the basis of cross-agglutination tests using serogroup-specific hyperimmune rabbit sera in the microscopic agglutination test (MAT). After genome sequencing, isolate identity was subsequently confirmed using the core-genome multilocus sequence typing (cgMLST) scheme based on 545 highly conserved loci, developed by Guglielmini et al. (2019) and implemented on the Institut Pasteur webstite BIGSdb-Pasteur (https://bigsdb.pasteur.fr/).

### 2.2 Restriction endonuclease analysis

To enable the preliminary identification of selected isolates as belonging to the *L. interrogans* serovar Hardjo type Prajitno, we performed restriction endonuclease analysis (REA) following established protocols. Genomic DNA was extracted from 4 *L. interrogans* isolates (KR40, KR84, KR85, and N116) using the QIAamp DNA Mini Kit (Qiagen) Genomic, according to the manufacturer’s instructions. For comparative purposes, genomic DNA from two Hardjo strains were also included: *L. interrogans* serovar Hardjo strain Hardjoprajitno and *L. borgpetersenii* serovar Hardjo strain KR39 (BioProject: PRJNA828006).

DNA samples were digested with the restriction enzyme EcoRI (Thermo Fisher Scientific), using 1 µg of DNA and 10 units of enzyme in a 20 µL reaction volume, incubated at 37 °C for 1 h. The resulting DNA fragments were separated by electrophoresis on a 0.6% agarose gel in 1× TBE buffer at 70 V for 21 h. Gels were stained with ethidium bromide and visualised under UV light using a GelDoc imaging system (G:BOX Chemi system (Syngene) equipped with GeneSys/GeneTools software). Banding patterns were analysed visually and compared with the reference strains following the method described by Ellis et al. (1991). Strains exhibiting REA profiles consistent with the Hardjoprajitno reference were considered to belong to the *L. interrogans* sv. Hardjo (type Prajitno) lineage.

### 2.3 Whole genome sequencing and annotation

Genomic DNA was isolated from bacterial cultures using the Genomic Mini AX Bacteria kit (A&A Biotechnology, Poland) and quantified using the NanoDrop 2000 and Qubit systems (Thermo Scientific). Genomic DNA fragmentation was verified by agarose gel electrophoresis. Illumina sequencing libraries for strains under investigation were prepared from 200 ng total DNA according to the manufacturer’s instructions with the Lotus DNA Library Prep Kit (Integrated DNA Technologies, Inc.). Sequencing was performed using single-end 75 bp runs on an Illumina NextSeq 550 platform with a minimum of 40x genome coverage. Oxford Nanopore sequencing libraries, due to objective reasons, were generated only for the KR40 and N116 strains, and were prepared with 3 µg of total DNA using the Ligation Sequencing Kit (SQK-LSK110; Oxford Nanopore Technologies, Oxford, UK) according to the manufacturer’s protocol. The libraries were loaded onto R10.3 flow cells and sequenced on the MinION Mk1C system (Oxford Nanopore Technologies). Basecalling was carried out with Guppy (v5.0.11+2b6dbff) using the super-accuracy model.

For KR84 and KR85 strains, Illumina raw sequencing reads were evaluated for quality using FastQC v0.11.9 (Andrews, 2010) and filtered using TrimGalore software v0.6.10 (Krueger, 2021). Filtering was done through the removal of adapter sequences and trimming of low-quality read ends. Filtered reads were assembled into contigs using Shovill v1.1.0 (https://github.com/tseemann/shovill), an ultra-fast implementation of the SPAdes v3.14.0 algorithm (Nurk et al., 2013). The resulting contigs were polished using Pilon v1.23 (Walker et al., 2014). Contigs statistics were retrieved using QUAST software v5.0.2 (Mikheenko et al., 2018). Scaffolding was carried out by CSAR-web (Chen and Lu, 2018), using the closest available complete genome for the analysis at the time of analysis, determined by 16S rRNA analysis: *L. interrogans* serovar Hardjo strain Norma (reference assembly: GCA_001293065.1). Genome sequences were annotated using the NCBI Prokaryotic Genome Annotation Pipeline (PGAP) with the best-placed reference protein set (GeneMarkS-2+) v6.0. The assemblies were deposited in GenBank under BioProject PRJNA809530 (KR84 assembly: GCA_022436545.1; KR85 assembly: GCA_022436605.1).

For KR40 and N116 strains (hybrid assemblies) raw Illumina reads were qualified by FastQC for quality and trimmed using Flexbar v3.5.0 (Dodt et al., 2012), removing adapter sequences and trimming low-quality read ends. Nanopore reads were trimmed using Fastp v0.23.2 (Chen et al., 2018), and adapter sequences were removed using Porechop v0.2.4 (Wick et al., 2017) and filtered with Filtlong v0.2.1 (https://github.com/rrwick/Filtlong), setting a minimum nucleotide length of 500 bp. Low-quality read ends were trimmed using Fastp. Filtered Illumina and Nanopore reads were assembled *de novo* with a hybrid strategy using Unicycler v0.5.0 (Wick et al., 2017) with default parameters. The obtained assemblies were annotated using the NCBI Prokaryotic Genome Annotation Pipeline (PGAP) with best-placed reference protein set (GeneMarkS-2+) v6.1 (strain N116) and v6.2 (KR40). Assembly was deposited in the GenBank under the BioProject number PRJNA828004 (assembly: GCA_023158895.3) and PRJNA828002 (assembly: GCA_023515975.1) for KR40 and N116 respectively.

### 2.4 Comparative genomics and phylogenetic analyses

Genomic relatedness of analysed strains was compared with 19 publicly available *L. interrogans* genomes from the NCBI database. All genomes were selected based on complete assembly status, to ensure consistent genome quality and to avoid bias in downstream analyses. They represent a wide range of serovars, geographic regions, host species, and collection years (Supplementary Table S1). The only exception was made for 3 *L. interrogans* serovar Hardjo strains (str. KR84, KR85, and OV5) which were included despite being high-quality draft assemblies. This was due to the limited availability of complete genomes for serovar Hardjo in public databases at the time of study design. Excluding them would have significantly reduced the representativeness of the Hardjo group.

The relationship between the number of coding sequences (CDS) and total genomic length was assessed using Spearman’s rank correlation, due to non-normal distribution. The average nucleotide identity (ANI) was calculated using JSpecies with ANIm option (Richter et al., 2016). Variant calling analysis (VCA) was performed to identify point mutations and establish population and phylogenetic structure. Filtered sequences were mapped to the reference genome *L. interrogans* sv. Hardjo str. Norma (Ref Seq: GCF_001293065.1) using BWA v0.7.17 (Li, 2013). Variants were identified using Freebayes v1.3.6 (Garrison and Marth, 2012) and filtered by a quality score cut-off >30 and depth >10. Variants were classified as SNPs (single nucleotide polymorphisms), MNPs (multi-nucleotide polymorphisms), INDELs (insertions/deletions), or mixed variants, which comprised a combination of the above types. The effects of the variants (excluding upstream/downstream, 5′UTR/3′UTR, and intronic changes) were predicted with SnpEff (Cingolani, 2022). Additionally, each of the selected high-impact variants was manually examined to evaluate its phenotypic relevance by determining whether the nucleotide change altered the protein sequence or led to its complete absence. Population structure analysis was conducted with the ADMIXTURE programme (Alexander et al., 2009) for K values from 1 to 10, selecting the optimal K based on cross-validation error. Results were visualised in Clumpak (Kopelman et al., 2015). The phylogenetic tree was constructed using the IQ-TREE web server with the auto-detect best-fit model with 1,000 ultrafast bootstraps (Nguyen et al., 2015) and visualised with iTOL v5 (Letunic and Bork, 2021). Ortholog clusters of the entire set of protein sequences were established using OrthoFinder (v3.0.1) with default parameters (Emms and Kelly, 2019). To assess differences between Hardjo strains and non-Hardjo strains, genomes were divided into two groups: *L. interrogans* serovar Hardjo group (LiH) and *L. interrogans* serovar non-Hardjo group (Li-nH). Statistical differences between groups were assessed using Fisher’s Exact Test, which is appropriate for categorical data and small sample sizes. To account for multiple testing, the Bonferroni correction was applied by dividing 0.05 by the number of tests (separately for VCA analysis and ortholog clusters analysis) to establish the initial significance threshold. However, due to the limited sample size and the highly conservative nature of the Bonferroni method, which may lead to a high false-negative rate, we applied a relaxed threshold - filtering based on raw p-values <5 × 10^−6^ for VCA analysis and 1 × 10^−4^ for ortholog clusters analysis. All calculations were performed in R (v4.4.2).

### 2.5 *In silico* characterisation of genes and proteins

Genes and encoded proteins of interest were further searched in the UniProt database (Bateman et al., 2023) and the STRING database (Szklarczyk et al., 2023). Functional classification was established with the COG database using COGclassifier v1.0.5 (Tatusov et al., 2000). The CELLO programme v.2.5 (Yu et al., 2006), PSORT database v3.0.3 (Venus Lau et al., 2021), and SOSUI GramN programme v1.11 (Imai et al., 2008) were used to predict protein subcellular localisation with a majority voting strategy, as previously described (Techawiwattanaboon et al., 2023). Additionally, proteins that were predicted to be outer membrane, inner membrane or periplasmatic proteins by different programmes, without a majority voting strategy, were assigned to a newly created category labelled as ‘Membrane’. The SignalP 6.0 server (Teufel et al., 2022) was used to search for signal peptides. Proteins not detected by SignalP were further processed by the SecretomeP 2.0 (Bendtsen et al., 2005) to predict non-classical secreted proteins. To improve the prediction of spirochaetal lipoproteins, we additionally applied SpLiP tool (Setubal et al., 2006). All predictions were performed with default settings for Gram-negative bacteria. For genes and proteins lacking predicted information from the described procedures, we employed DeepFRI programme for functional annotation (Gligorijević et al., 2021). Genes and variants of interest were further categorized into those occurring outside the *rfb* locus and those within the *rfb* locus. The localisation of the *rfb* locus was determined based on the region spanning from the *MarR* regulatory gene to the *DASS* gene (Fouts et al., 2016; Ferreira et al., 2024). Probable genomic islands associated with horizontal gene transfer were predicted using the IslandViewer 4 platform (Bertelli et al., 2017). Gene localisation and COG categories were visualised using in-house R scripts (v4.4.2).

### 2.6 Biofilm quantification

To confirm the hypothesis that genetic changes could impact the capacity of Hardjo and non-Hardjo strains of *L. interrogans* to form biofilm, we evaluated biofilm formation *in vitro.* Biofilm formation by *L. interrogans* (serovars Hardjo, Copenhageni, and Bratislava) was evaluated using a static microtiter plate assay according to the protocol of Thibeaux et al. (2020a). Bacteria were grown in EMJH medium at 30 °C under static conditions to the exponential phase, adjusted to a final density of 1 × 10^6^ cells/mL, and seeded into sterile, flat-bottom 96-well polystyrene plates (150 µL per well). Plates were incubated at 30 °C for 21 days without medium replacement to allow for mature biofilm formation. After incubation, planktonic cells were gently removed, and biofilms were fixed with 4% paraformaldehyde for 30 min. Wells were then washed with PBS, stained with 0.1% crystal violet for 10 min, and washed again. Stained biofilm was solubilised using 30% acetic acid, and absorbance was measured at 570 nm using a microplate reader. Biofilm assays were performed in three independent experiments, each with triplicate wells per condition. Statistical comparisons were based on biological replicates (n = 3 per group per time point). Statistical significance between serovars at each time point was assessed using the Kruskal-Wallis test, followed by pairwise Mann-Whitney U tests with Bonferroni correction for multiple comparisons. A p-value <0.05 was considered statistically significant.

## 3 Results

### 3.1 Sample characterisation

**FIGURE 1:**
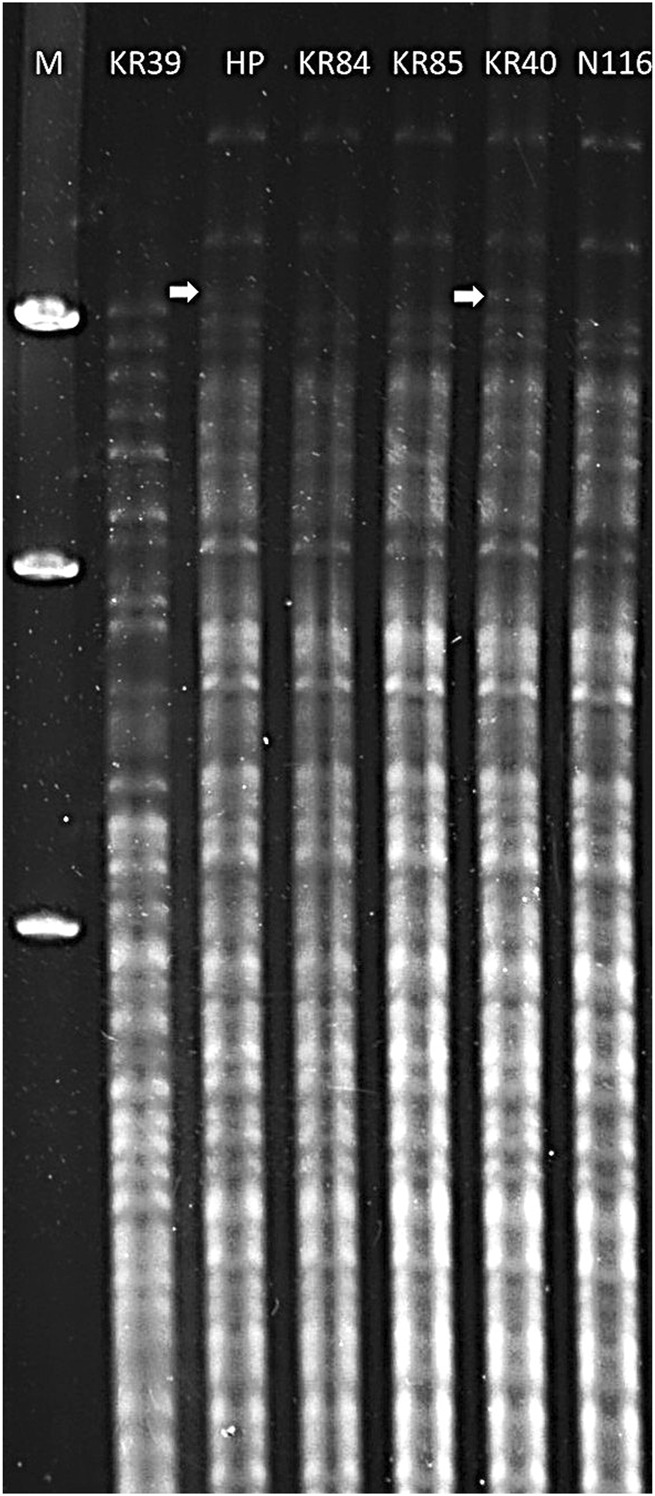
Restriction endonuclease analysis patterns of DNA from *Leptospira* strains digested with EcoRI. Lanes: 1 – Marker Lambda/Hind III (A&A Biotechnology); 2 *– L. borgpeter*senii sv. Hardjo str. KR39; 3 *– L. interrogans* sv. Hardjo str. Hardjoprajitno; 4 – *L. interrogans* serovar Hardjo str. KR84; 5 – *L. interrogans* sv. Hardjo str. KR85; 6 – *L. interrogans* sv. Hardjo str. KR40; 7 - *L. interrogans* sv. Hardjo str. N116; white arrow - presence of an additional high molecular weight band (∼9,416 bp).

Serological characterisation indicated that the tested field isolates belonged to the Sejroe serogroup (data not shown). For the preliminary identification of isolates as members of the *L. interrogans* serovar Hardjo, we applied the restriction endonuclease method (REA). Six strains belonging to serovar Hardjo were included in the analysis. The results revealed that DNA isolated from KR84, KR85, KR40, and N116 strains (Figure 1, lanes 4, 5, 6, 7) showed similar restriction patterns, consistent with that of the reference genome *L. interrogans* sv. Hardjo str. Hardjoprajitno (Figure 1, lane 3). The pattern for *L. interrogans* sv. Hardjo differed from that of *L. borgpetersenii* sv. Hardjo (Figure 1, lane 2). A minor difference was observed in the absence of an extra high molecular weight band of approximately 9,416 bp (Figure 1, white arrow) in the KR84, KR85, and N116 isolates (Figure 1, lanes 4, 5, and 7) compared with strains KR40 and Hardjoprajitno (Figure 1, lanes 3 and 6). Consistent with these REA findings, cgMLST analysis ultimately confirmed the taxonomic assignment - all four isolates grouped in cluster 40 (clonal group 19) of the cgMLST scheme, together with other representatives of *L. interrogans* serogroup Sejroe serovar Hardjo. The curated records are publicly available in BIGSdb-Pasteur (https://bigsdb.pasteur.fr/leptospira) under IDs: 1234 (KR85), 1271 (N116), 1272 (KR40) and 1336 (KR84).

### 3.2 Assembly and annotation of the sequenced genomes

Sequencing statistics for *L. interrogans* strains under study are summarised in Supplementary Table S2. The generated sequencing reads provided genome coverage of approximately 42x for assemblies based solely on Illumina reads (strains KR84 and KR85) and over 114x for hybrid assemblies. Using hybrid assembly approaches, complete genomes consisting of five and four circular contigs were obtained for strains N116 and KR40, respectively. For strains KR84 and KR85, draft whole-genome sequences consisted of 177 and 186 contigs, respectively. After scaffolding against the genome of *L. interrogans* serovar Hardjo str. Norma, nine scaffolds were generated for KR84 and seven scaffolds for KR85. Hybrid assemblies could not be obtained for strains KR84 and KR85 in this study. The genome size and number of predicted genes suggest, however, a high level of the completeness of their genomes, even in non-hybrid assemblies. The high sequencing coverage enabled a nearly complete representation of the genomic content, with only homologous or repetitive regions potentially preventing full assembly into continuous, circularised sequences (str. KR84, KR85).

Table 1 presents the quality control parameters for the four studied strains of *L. interrogans,* while Table 2 summarises their genome annotation parameters. The annotated genomes displayed a similar number of detected genes, with an average of 3,930 and an interquartile range (IQR) of 111.5; the gene count was slightly higher in the hybrid assemblies. On average, 96% of the genes were protein-coding. Each genome contained between 3 (for whole genome sequences of KR84 and KR85) and 5 complete rRNAs (for complete genome sequences for genomes KR40 and N116), 37 tRNAs, and approximately 156 pseudogenes (IQR: 7.75). The genomic G + C content ranged from 34.96% to 35.05%.

**TABLE 1:** Assembly quality control parameters for studied strains of *Leptospira interrogans* sv. Hardjo.

Strain	Total ungapped length	Number of scaffolds	Scaffold N50	Scaffold L50	Number of contigs	Contig N50	Contig L50	Largest contig	# Ns per 100 kbp
N116	4,847,508	-	-	-	5	4,312,952	1	4,312,952	0.00
KR40	4,793,656	-	-	-	4	4,315,670	1	4,315,670	0.00
KR84	4,604,145	9	4,252,347	1	177	43,336	35	93,543	363.56
KR85	4,609,556	7	4,296,696	1	186	46,778	34	79,011	386.82

**TABLE 2:** Genome annotation parameters for studied strains of *Leptospira interrogans* sv. Hardjo

Strain	Genes (total)	Genes (coding)	Coding genes (%)	CDSs (total)	CDSs (with protein)	CDS with protein (%)	Complete rRNAs	tRNAs	ncRNAs	Pseudogenes (total)	CDSs (without protein)
N 116	3,992	3,787	94.87	3,948	3,787	95.92	5	37	2	161	161
KR40	3,979	3,776	94.90	3,935	3,776	95.96	5	37	2	159	159
KR84	3,865	3,672	95.01	3,823	3,672	96.05	3	37	2	151	151
KR85	3,883	3,689	95.04	3,841	3,689	96.04	3	37	2	152	152

### 3.3 Comparative genomics

Genetic analysis was conducted on the four genomes obtained during this study and on an additional 19 genomes of *L. interrogans,* selected from the publicly available NCBI database (Supplementary Table S1). The genomic sequence length ranged from 4,630,592 to 5,349,767 bp with an average GC content of 35.01%. The total number of genes per genome ranged from 3,762 to 5,312. The number of coding sequences had a positive Spearman’s correlation of 0.734 (p = 6.73 × 10^−5^) with the genomic length.

ANI analysis provided a comparative genomic relatedness between various serovars and strains of *L. interrogans* (Supplementary Figure S1). The results from the JSpecies analysis revealed a high ANI percentage, ranging from 98.15% to 99.98% between different strains in our study. Serovar Hardjo displayed ANI values close to 100% among its strains. Lower ANI values, as shown in the heatmap in blue, suggest increased genetic distance and greater diversity, especially within the *L. interrogans* sv. Canicola and sv. Manilae, signifying potential genomic variability or divergence from other serovars.

### 3.4 Population structure and phylogenetic analysis

**FIGURE 2:**
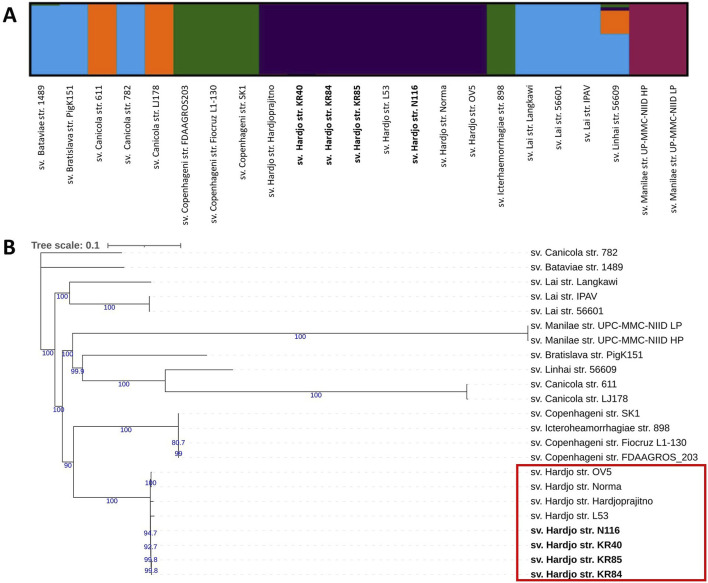
Population structure and phylogenetic relationships of *Leptospira interrogans* strains show a clear separation of serovar Hardjo. **(A)** Population structure analysis of *L. interrogans* strains using ADMIXTURE. Results are shown for K = 5; **(B)** Maximum likelihood SNP-based phylogenetic tree of *L. interrogans* with 1,000 ultrafast bootstrap replicates with TVM + F substitution model. Genomes obtained during this study were bolded. Only bootstrap values greater than 60 were shown (blue colour).

Population structure analysis and phylogenetic reconstruction were performed based on the output of variant calling analysis (VCA). Population structure analysis (Figure 2A), conducted using the ADMIXTURE programme, indicated an optimal K value of 5 (log-likelihood = −344427.89, CV error: 0.77). The analysis separated all sv. Hardjo strains from other serovars. Since traditional phylogenetic markers such as 16S rRNA lack the resolution to distinguish between closely related serovars within the same species, we employed SNP-based analysis to achieve a higher level of phylogenetic resolution for the analysed *L. interrogans* strains. A phylogenetic tree was constructed using IqTree with TVM + F substitution model and 1,000 ultrafast bootstrap option (Figure 2B). We obtained strong bootstrap support, particularly for internal nodes connecting major clades. The phylogenetic tree, with some exceptions, revealed serovar-specific branches. The topology of the tree suggest that serovar Hardjo is the most closely related to members of the Icterohaemorrhagiae serogroup. Among all Hardjo representatives, short phylogenetic branches indicate limited genetic diversity.

### 3.5 Variant calling analysis and identification of Hardjo-specific effects

For the purposes of this study, VCA using *L. interrogans* sv. Hardjo str. Norma as the reference genome was chosen. Approximately 99.4%–99.9% of the Hardjo reads were successfully aligned to the reference genome, whereas alignment rates for reads from other serovars varied from 87.9% for sv. Canicola to 96.6% for sv. Copenhageni.

Table 3 provides a summary of all high-quality variants detected across the dataset, as well as the subset of statistically significant differences between LiH and Li-nH groups. In total, the analysis revealed 99,656 genetic variants and 99,771 predicted effects on gene and their encoded proteins. Out of these, 10,266 variants and 10,724 effects were identified as statistically significant as differentiating between the groups, including 6,299 in the coding region and 4,425 in the non-coding region. Statistically significant variants were primarily located outside the *rfb* locus, although some conserved segments within this region also exhibited polymorphisms. Among genetic variants, SNPs were the most abundant. Overall, the ratio of non-synonymous to synonymous changes (dN/dS) was 0.4336, and was similar for statistically significant differences between the Hardjo and non-Hardjo groups, with a value of 0.4256.

**TABLE 3:** The statistics of VCA for results with quality >30 and deep >10. The column “All” presents the total number of all observed variants passing quality thresholds. The two subsequent columns show statistically significant differences between the LiH group and the Li-nH group (p-values <5 × 10^−6^, calculated using Fisher’s exact test with a relaxed Bonferroni-derived cut-off). Results are further divided by genomic region (coding vs. non-coding) and by their location relative to the *rfb* locus.

	All	p < 5 × 10^−6^	
		Outside *rfb* locus	Inside *rfb* locus
Number of variants by type			
SNP	81,403	7,165	51
MNP	10,130	1,789	18
Insertion	2,872	424	1
Deletion	3,442	370	0
Mixed	1,809	448	0
Number of effects by region			
Coding region	62,098	6,243	56
Non-coding region	37,662	4,411	14
Transcript	11	0	0

**FIGURE 3:**
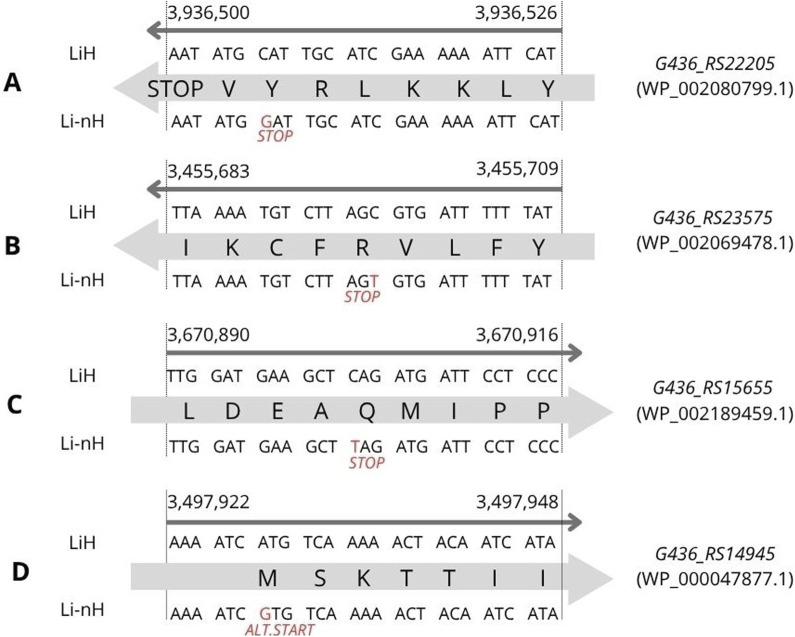
SNP variants identified through variant calling analysis were associated with phenotypic differences observed between the LiH and Li-nH groups (p < 5 × 10^−6^, calculated using Fisher’s exact test with a relaxed Bonferroni-derived cut-off). Each mutation is annotated based on its location on either the forward or reverse strand. **(A)** Premature stop codon in *G436_RS22205* gene; **(B)** Premature stop codon in *G436_RS23575* gene; **(C)** Premature stop codon in *G436_RS15655*; **(D)** Alternative start codon in *G436_RS14945* gene.

We identified four statistically significant high-impact SNPs with predicted effects on the corresponding protein sequences. One of them, was located in the *G436_RS22205* gene (p = 8.7 × 10^−12^), involving a cytosine (C) to guanine (G) substitution at position 3,936,506 on chromosome I (Figure 3A). This mutation introduces a premature stop codon, resulting in the absence of the encoded protein (WP_002080799.1) in non-Hardjo serovars. According to UniProt, the protein is annotated as a mobile element protein (A0A0M4NZF1), showing 100% identity with a PF07600 domain-containing sequence. Two additional premature stop codons were detected with lower statistical significance (p = 2.8 × 10^−6^). The first was located at position 3,455,697, involving a cytosine (C) to thymine (T) substitution in gene *G436_RS23575* (Figure 3B), affecting a hypothetical protein (WP_002069478.1), suggested by DeepFRI to localise in the cytoplasm and potentially participate in general metabolic processes. The second occurred at position 3,670,902, involving a cytosine (C) to thymine (T) substitution in gene *G436_RS15655* (Figure 3C), encoding the CRISPR-associated helicase Cas3 (WP_002189459.1), which appears as a pseudogene or truncated protein in Li-nH group. Additionally, we identified a SNP in *G436_RS14945* (Figure 3D), where an adenine (A) to guanine (G) substitution at position 3,497,928 alters a start codon from ATG to GTG in non-Hardjo strains, affecting translation initiation for the thiazole kinase ThiM (WP_002188296.1; p = 1.0 × 10^−9^).

**FIGURE 4:**
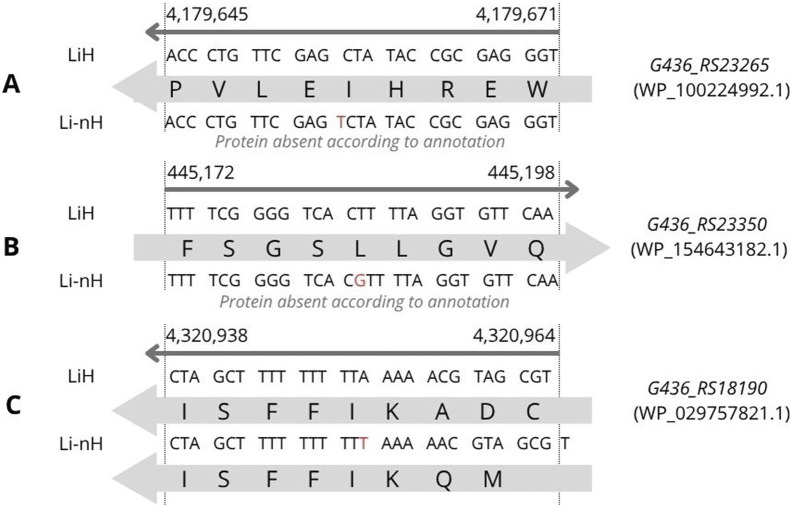
INDEL variants identified through variant calling analysis were associated with phenotypic differences observed between the LiH and Li-nH groups (p < 5 × 10^−6^, calculated using Fisher’s exact test with a relaxed Bonferroni-derived cut-off). Each mutation is annotated based on its location on either the forward or reverse strand. **(A)** Loss of coding sequence due to an insertion in gene *G436_RS23265*; **(B)** Loss of coding sequence due to an insertion in gene *G436_RS23350*; **(C)** Frameshift caused by an insertion in gene *G436_RS18190.*

In addition, three INDELs were identified with statistically significant frequency differences between LiH and Li-nH groups, each predicted to affect protein sequence. Two of them resulted in the complete loss of protein-coding sequence in the Li-nH group. The first, located in *G436_RS23265* (Figure 4A), involves a thymine (T) insertion leading to the absence of protein WP_100224992.1 (p = 8.7 × 10^−12^). The second, in *G436_RS23350* (Figure 4B), results from a guanine (G) insertion and frameshift, leading to disruption of WP_154643182.1 coding sequence in Li-nH (p = 1.0 × 10^−9^). Both proteins are hypothetical and predicted to localize to the cytoplasm, with putative roles in metabolic processes, as predicted by DeepFRI. The third INDEL, located in *G436_RS18190* (Figure 4C), modifies the N-terminal region of the FecR family protein WP_029757821.1, shortening it from 389 to 385 amino acids in non-Hardjo strains (p = 8.7 × 10^−12^). FecR proteins are implicated in iron uptake via citrate transport, though the functional consequences of this truncation remain unclear.

We also identified statistically significant, high-impact mutations, including one start-loss variant, six stop-gain variants, 17 stop-loss variants, 25 frameshift variants, and 13 complex variants occurring in Hardjo pseudogenes (Supplementary Table S3). According to the RefSeq annotation, *L. interrogans* sv. Hardjo str. Norma has a total of 184 pseudogenes, which constitute 3.7% of all genes. A number of pseudogenes in each newly sequenced genome is shown in Table 2, and varies from 151 to 161.

In total, we identified 1,335 significant missense variants, 159 of which were located in pseudogenes. The remaining 1,176 variants were mapped to 954 distinct genes. Particular attention was given to genes encoding predicted outer membrane proteins (OMPs), due to their surface localisation and possible interaction with the host environment. To identify proteins with N-terminal signal peptides and signal peptidase cleavage sites, SignalP 6.0 was used. Among the 954 genes analysed, 191 were predicted to encode proteins with secretion signal sequences*.* Of these, 119 had secretory signal peptides transported by the Sec translocon and cleaved by Signal Peptidase I (SP); 66 had lipoprotein signal peptides transported by the Sec translocon and cleaved by Signal Peptidase II (LIPO); four contained Tat signal peptides transported by the Tat translocon and cleaved by Signal Peptidase I (TAT); one had a Tat lipoprotein signal peptide transported by the Tat translocon and cleaved by Signal Peptidase II (TATLIPO); and one had pilin and pilin-like signal peptides transported by the Sec translocon and cleaved by Signal Peptidase III (PilD/PibD) (PILIN). Due to the plasticity of spirochaetal lipobox sequences, SpLiP algorithm, a tool optimised for spirochaetes, was used. 55 proteins were predicted by SpLip as probable lipoproteins and 12 as possible lipoproteins. 80 proteins were predicted by SecretomeP to be exported through the non-classical protein secretion pathway. To further predict protein subcellular localisation, we applied the CELLO programme, the PSORT database, and SOSUI GramN programme applying a majority-voting strategy to enhance prediction accuracy. 29 proteins were predicted as ‘Extracellular’, 49 as ‘Outer membrane’, 130 as ‘Inner membrane’, 12 as ‘Periplasmic’, whereas 56 were assigned to general category ‘Membrane’ (Supplementary Table S4).

### 3.6 Identification of unique cluster of orthologues

**FIGURE 5:**
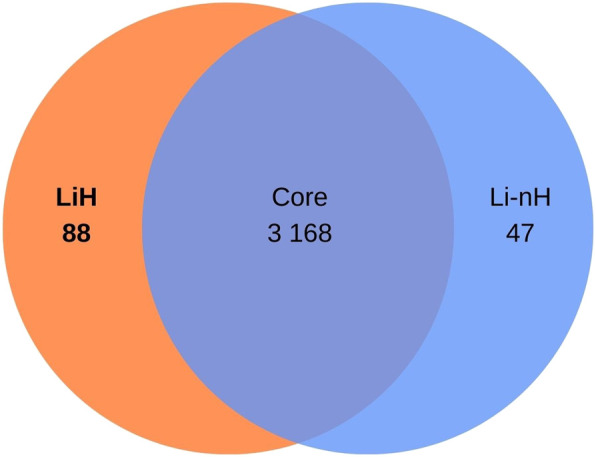
Venn diagram illustrating the distribution of orthogroups identified in *Leptospira interrogans*. A total of 3 168 orthogroups were classified as core genes. Additionally, 88 orthogroups were identified as specific to the Hardjo serovar (LiH), while 47 orthogroups were specific to other serovars (Li-nH), being absent in the Hardjo serovar (p < 1 × 10^−4^; calculated using Fisher’s exact test with a relaxed Bonferroni-derived cut-off).

The OrthoFinder programme identified 4,326 orthologous groups across the analysed genomes. The pangenome of *L. interrogans* comprised 3,168 core orthologous gene clusters, defined as those present in at least 95% of the analysed genomes. 88 orthogroups were classified as unique to the Hardjo serovar (LiH). Conversely, 47 orthogroups were predominantly present in the non-Hardjo group (Li-nH) (Figure 5). A full list of LiH-specific proteins, along with predicted localisation in the genome, potential localisation in the cell compartment, potential prediction of non-classical protein secretion, and prediction of lipoproteins is in Table 4 (for genes located outside the *rfb* locus) and Supplementary Table S5 (for genes located within the *rfb* locus).

**TABLE 4:** A complete list of orthologues proteins specific to LiH group (p < 1 × 10^−4^; calculated using Fisher’s exact test with a relaxed Bonferroni-derived cut-off) outside *rfb* locus, along with potential subcellular localisation (Subcell. Loc.) determined using a combination of methods, including CELLO, PSORTdb, and the SOSUI, SpLip for lipoprotein prediction, as well as prediction of non-classical protein secretion type by SecretomeP (SecP). Additionally, the table groups genes based on their location within defined genomic regions (Gen. region) and predicted genomic islands (GI) as identified by IslandViewer 4.

Protein id	Protein name	Subcell. Loc.	SecP/SpLip	GI	Gen. region
Outside *rfb* locus					
WP_001185452.1	Mobile element protein	Unknown	SecP+		I
WP_000044296.1	Toxin-antitoxin system, antitoxin component, ribbon-helix-helix domain protein	Cytoplasmic	SecP+		
WP_000014356.1	Type II toxin-antitoxin system VapC family toxin	Unknown			
WP_002188771.1	Hypothetical protein	Unknown			
WP_002074899.1	Hypothetical protein	Unknown			
WP_001127181.1	Mobile element protein	Cytoplasmic	SecP+		
WP_002188784.1	Hypothetical protein	Cytoplasmic			
WP_000348397.1	Hypothetical protein	Cytoplasmic			
WP_025177720.1	Hypothetical protein	Inner Membrane			
WP_025177718.1	Hypothetical protein	Unknown			
WP_025177717.1	Hypothetical protein	Cytoplasmic			
WP_002188765.1	Toxin-antitoxin system, antitoxin component, ribbon-helix-helix domain protein	Cytoplasmic			
WP_002188794.1	Hypothetical protein	Cytoplasmic			
WP_002189359.1	Hypothetical protein	Cytoplasmic		+	II
WP_017853759.1	Hypothetical protein	Cytoplasmic		+	
WP_002189216.1	IS1595 family transposase	Cytoplasmic		+	
WP_002189253.1	Hypothetical protein	Cytoplasmic		+	
WP_002189271.1	Sce7726 family protein	Cytoplasmic		+	
WP_002189307.1	Sce7725 family protein	Cytoplasmic		+	
WP_025177767.1	Hypothetical protein	Cytoplasmic		+	
WP_017853768.1	Hypothetical protein	Cytoplasmic	SecP+	+	
WP_002189420.1	Hypothetical protein	Cytoplasmic		+	
WP_223813948.1	Helix-turn-helix transcriptional regulator	Cytoplasmic		+	
WP_002189209.1	Hypothetical protein	Cytoplasmic		+	
WP_033108691.1	Hypothetical protein	Inner Membrane		+	
WP_002101393.1	Hypothetical protein	Cytoplasmic		+	
WP_000024079.1	Hypothetical protein	Cytoplasmic		+	
WP_002189393.1	Hypothetical protein	Cytoplasmic		+	
WP_002189380.1	Hypothetical protein	Cytoplasmic		+	
WP_017854057.1	Hypothetical protein	Cytoplasmic		+	
WP_002189396.1	Hypothetical protein	Cytoplasmic		+	
WP_002189105.1	Hypothetical protein	Cytoplasmic			III
WP_001147350.1	HEAT repeat domain-containing protein	Cytoplasmic			
WP_002189053.1	Hypothetical protein	Inner Membrane			
WP_000730340.1	Hypothetical protein	Cytoplasmic			
WP_002119210.1	Hypothetical protein	Cytoplasmic			
WP_025177732.1	Hypothetical protein	Cytoplasmic			
WP_000905909.1	IS3 family transposase	Cytoplasmic			
WP_002074414.1	Transposase	Cytoplasmic			
WP_000937014.1	Hypothetical protein	Cytoplasmic			
WP_002188149.1	Hypothetical protein	Cytoplasmic			
WP_002189012.1	Putative lipoprotein	Cytoplasmic		+	
WP_000240615.1	Hypothetical protein	Cytoplasmic		+	
WP_002187625.1	Hypothetical protein	Cytoplasmic		+	
WP_002187597.1	TIR domain-containing protein	Cytoplasmic		+	
WP_000446747.1	Hypothetical protein	Cytoplasmic	SecP+		
WP_002188813.1	Leucine-rich repeat domain-containing protein	Cytoplasmic			
WP_000639168.1	Hypothetical protein	Cytoplasmic		+	
WP_000881563.1	Toxin-antitoxin system, antitoxin component, ribbon-helix-helix domain protein	Cytoplasmic			
WP_002188763.1	Hypothetical protein	Cytoplasmic			
WP_020781169.1	Hypothetical protein	Unknown	SecP+		
WP_002109126.1	N-6 DNA methylase	Unknown			
WP_154643182.1	Hypothetical protein	Unknown			
WP_000288518.1	Guanylate cyclase domain-containing protein/Adenylate/guanylate cyclase catalytic domain protein	Unknown		+	
WP_001973742.1	Hypothetical protein	Unknown			
WP_002080799.1	Mobile element protein	Unknown	SecP+		
WP_002187702.1	PF06803 family protein	Cytoplasmic			
WP_001208091.1	HEPN domain-containing protein	Cytoplasmic		+	
WP_002187518.1	Hypothetical protein	Cytoplasmic		+	

**FIGURE 6:**
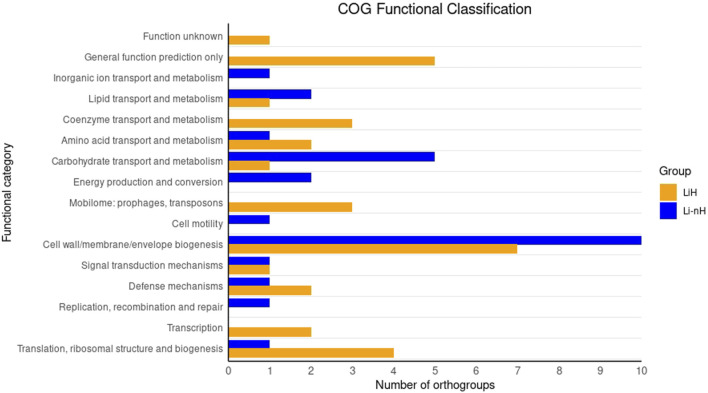
COG functional classification of orthgroups unique to LiH and Li-nH groups.

The analysis of orthogroups specific to Hardjo and non-Hardjo serovars, revealed distinct functional profiles, as classified by COG categories. In the LiH group, 36.36% (32/88) of sequences were assigned to COG functional categories, compared to 55.32% (26/47) in the Li-nH group (Figure 6). Among the categories, ‘Cell wall/membrane/envelope biogenesis’ displayed the most significant differences.

Regarding the predicted subcellular localisation, 70 proteins were classified as ‘Cytoplasmic’, 6 as ‘Inner Membrane’ and 1 as ‘Outer Membrane’. None were predicted by SignalP whereas seven were identified by SecretomeP as candidates for non-classical secretion. However, all were classified as cytoplasmic or of unknown localisation, mostly representing hypothetical or mobile element proteins, often secreted via type IV systems in Gram-negative bacteria (Waksman, 2019). One protein (WP_000808306.1) was predicted as probable lipoprotein by SpLip.

**FIGURE 7:**
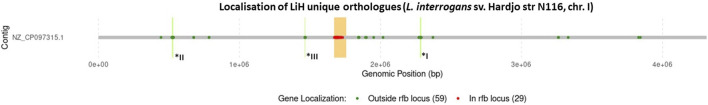
Localisation of genes encoding orhtogroups unique to sv. Hardjo on example of str. N 116. The *rfb* locus was marked in orange, with genes located within this region shown in red, and those outside it in green. Number of genes in each group is provided in parentheses. Additionally, genomic regions I–III, corresponding to positions I: 2,280,254–2,289,105, II: 521,196–532,002, and III: 1,463,770–1,468,634, are marked in bright green and labelled with asterisks.

As *rfb* locus is known for its high variability across *Leptospira* serogroups and serovars, we examined the localisation of LiH-specific orthogroups with respect to this region and visualised their distribution in the reference genome of strain N116 (Figure 7). Subsequent analyses focused primarily on the LiH-specific genes located outside the *rfb* locus. Of the 88 LiH-specific genes, 59 were located outside the *rfb* locus (Table 4). 37 of them were annotated as hypothetical proteins The observed clustering of several genes prompted us to assess their presence within putative genomic islands (GIs) using IslandViewer 4. Three large genomic regions enriched in LiH-specific genes were identified. The first region (positions 2,280,254–2,289,105 bp in str. N116) included genes for eight hypothetical proteins, two mobile element proteins (WP_001185452.1, WP_001127181.1), and three proteins associated with toxin-antitoxin systems (WP_000044296.1, WP_000014356.1, WP_002188765.1). The GC content of this region (39.96%) is noticeably higher than the average genomic GC content of *L. interrogans* strain N116 (35.05%). A second region (positions 521,196–532,505 bp; GC content 30.29%) comprised fourteen hypothetical proteins, an IS1595 transposase (WP_002189216.1), and two Sce7726 and Sce7725 family proteins (WP_002189271.1 and WP_002189307.1, respectively). Third region (1,463,770–1,468,634 bp) contained mainly hypotethical proteins (GC content 30.98%). In these regions, several interspersed genes were not classified as unique to *L. interrogans* sv. Hardjo. Additionally, large intergenic distances between some of the genes suggest that these clusters are unlikely to form single operon. While the first region is located entirely on the same strand, the second and third region span both DNA strands. Notably, only the second region was predicted as a genomic island by IslandViewer 4, whereas the first region was not.

Beyond these defined regions, we also detected several LiH-specific genes that either formed small local groupings or were scattered throughout the genome without consistent clustering. Among them were genes encoding a toxin-antitoxin system component (WP_000881563.1), a Toll/Interleukin-1 receptor domain containing protein (WP_002187597.1), a guanylate cyclase domain-containing protein (WP_000288518.1), and a leucine-rich repeat domain-containing protein (WP_002188813.1), positioned near the gene encoding the flagellar motor switch protein FliG. Of note, IslandViewer 4 predicted that some of these individual genes are located within genomic islands (Table 4).

### 3.7 Biofilm formation

**FIGURE 8:**
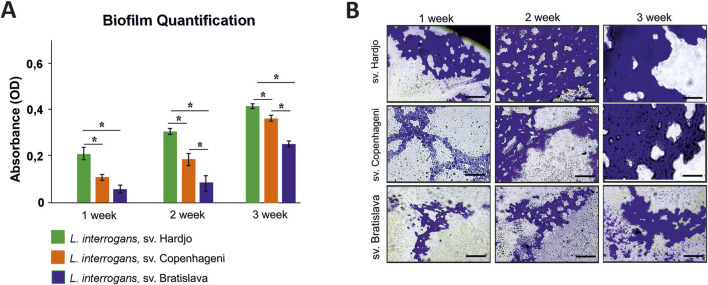
Biofilm formation by *Leptospira interrogans* serovars Hardjo, Copenhageni, and Bratislava over a 3-week incubation period. **(A)** Quantification of biofilm biomass by crystal violet staining expressed as optical density (OD) values measured at 570 nm. Bars represent mean ± SD (n = 3). Asterisks indicate statistically significant differences (p < 0.05); **(B)** Representative phase-contrast micrographs of biofilms stained with crystal violet. Scale bar 200 µm.

Crystal violet measurements revealed a clear time-dependent increase in biofilm biomass for all 3 *L. interrogans* serovars (Hardjo, Copenhageni, Bratislava) over the 21-day static incubation (Figure 8A). Serovar Hardjo formed significantly more biofilm than the other two serovars at each time point (p < 0.05), with absorbance values markedly higher by week 3. Serovar Copenhageni exhibited an intermediate biofilm production, whereas Bratislava consistently showed the lowest values. Microscopic examination mirrored these results: phase-contrast images (Figure 8B) demonstrated more extensive and confluent biofilm architecture for Hardjo, especially evident after 3 weeks, while Copenhageni and Bratislava produced progressively less dense microcolonies.

## 4 Discussion

The study examined the genomic features of *L. interrogans* serovar Hardjo isolates from Europe using a whole-genome sequencing approach, and revealed a high degree of sequence conservation, consistent with limited genetic variation previously described for host-adapted serovars. Although not all of the analysed isolates originated from cattle, it is important to note that *Leptospira* serovars, including Hardjo, are capable of causing incidental infections in non-maintenance hosts. These incidental infections often present with more pronounced clinical symptoms, increasing the likelihood of detection and subsequent isolation (Ellis, 2015). As such, even isolates obtained from incidental hosts may accurately reflect the genetic characteristics of strains circulating within the animal acting as maintenance host population and the broader environment, providing valuable insights into pathogen diversity.

The use of restriction endonuclease analysis (REA) enabled the preliminary identification of selected isolates as belonging to the *L. interrogans* serovar Hardjo (Figure 1), while whole-genome sequencing enabled in-depth comparative analysis with other *L. interrogans* serovars. This approach confirmed a high degree of genomic similarity among Hardjo isolates, with average nucleotide identity (ANI) values close to 100% between Hardjo strains (Supplementary Figure S1), confirming minimal divergence - potentially resulting from long-term infection within a maintenance host.

Using the VCA output, we performed population structure analysis (Figure 2A) and constructed a phylogenetic tree (Figure 2B). Both approaches demonstrated a distinct clustering pattern for serovar Hardjo, clearly separating it from other serovars. This may reflect lineage-specific adaptations that facilitate host association and prolonged colonisation of the reproductive tract in cattle and sheep. The Hardjo clade is most closely related to the serogroup Icterohaemorrhagiae clade (sv. Copenhageni and Icterohaemorrhagiae), which may confirm a previously proposed hypothesis that *L. interrogans* sv. Hardjo originated through horizontal gene transfer (HGT) from *L. borgpetersenii* sv. Hardjo to a strain of *L. interrogans* sv. Copenhageni (de la Peña-Moctezuma et al., 1999; Llanes et al., 2016). Therefore, the observed phylogenetic structure reinforces the value of genome-wide SNP analysis as a high-resolution tool for delineating *Leptospira* strains and resolving their evolutionary relationships. Such approaches can illuminate the intricate genetic structures within bacterial populations of various genera, thereby supporting phylogenetic studies vital for understanding pathogenicity and epidemiology (Filliol et al., 2006; Girault et al., 2014). For instance, SNP-based genomic analysis of *Streptococcus pyogenes* populations revealed distinct phylogenetic structures associated with niche-specific infections, such as skin disease and pharyngitis-induced acute rheumatic fever (Bao et al., 2016).

To better understand the genetic basis of this divergence, we next examined specific variants differentiating serovar Hardjo from other *L. interrogans* serovars. Among statistically significant changes, we identified four high-impact SNPs and three INDELs predicted to affect protein-coding sequences potentially relevant to host adaptation. Particularly noteworthy are the differences found in the gene encoding the CRISPR-associated helicase/endonuclease Cas3, and in the *thiM* gene.

The variant identified in *G436_RS15655* (Figure 3C), encoding the CRISPR-associated helicase/endonuclease Cas3 (WP_002189459.1), affected gene integrity across non-Hardjo serovars. In Hardjo strains, the gene was present in a complete, presumably functional form, whereas in other serovars it appeared truncated, likely leading to protein shortening or pseudogenization. CRISPR-Cas systems, including Cas3, have been found in both pathogenic and intermediate *Leptospira* species, with pathogenic strains often harbouring multiple types (e.g., subtype I-B and I-E) (Xiao et al., 2019). Cas3, a multi-domain protein with both helicase and nuclease activity, is known to play a role in protecting bacteria from mobile genetic elements. In other bacterial species, studies have implicated Cas3 in functions beyond immune defence, including possible roles in biofilm formation and virulence regulation (Rodrigues et al., 2023). Biofilm formation has been linked to reproductive tract colonisation and abortion, particularly in ruminants (Nesse et al., 2023; Araújo et al., 2024). In other *Leptospira* serovars, similar associations have been observed (Brihuega et al., 2012). In *Leptospira*, studies have proposed a link between Cas3 and biofilm formation, suggesting a contribution to bacterial persistence under host-associated stress conditions (Prakash and Kumar, 2022). This raises the hypothesis that Cas3-mediated mechanisms might contribute to the pathogenicity of *L. interrogan*s sv. Hardjo in ruminant hosts, potentially supporting its adaptation and prolonged persistence within reproductive tissues. However, this role remains putative, and experimental validation is required to confirm Cas3’s involvement in host-specific infection dynamics. In line with this hypothesis, our *in vitro* assay showed that sv. Hardjo formed biofilm more rapidly and in greater amounts than sv. Copenhageni and sv. Bratislava over a 21-day period (Figure 8), suggesting a possible functional consequence of Cas3 variation in the context of tissue colonisation. Quantitative trends were consistent across both techniques, reinforcing the conclusion that serovar Hardjo establishes biofilm more efficiently under these experimental conditions. While this phenotype was observed under controlled laboratory conditions, it may be consistent with enhanced persistence capacity relevant to the reproductive environment; nonetheless, direct causality remains to be investigated.

Another relevant variant was detected in the *thiM* gene (Figure 3D), which encodes thiazole kinase (WP_000047877.1), and altered the start codon from ATG to GTG. In prokaryotes, the start codon is one of the major translation initiation determinants. The canonical AUG start codon exhibits higher translation efficiency compared to alternative codons, such as GUG (Belinky et al., 2017). In bacteria, thiamine biosynthesis predominantly occurs via the *de novo* pathway. However, an alternative salvage pathway exists to recycle thiazole, a key intermediate. This salvage pathway ensures continued thiamine production when *de novo* synthesis is limited (Jurgenson et al., 2009). In our dataset, the canonical ATG start codon was predominantly observed in *L. interrogans* sv. Hardjo strains, whereas a GTG codon was more common in non-Hardjo strains. This pattern may suggest strain-specific variation in translation efficiency of thiazole kinase. Although the precise phenotypic consequences remain to be confirmed, reduced translation efficiency could theoretically affect thiamine salvage capacity under nutrient-limited conditions. Moreover, ThiM has been considered a potential antibacterial target, as its inhibition could disrupt thiamine biosynthesis and bacterial survival (Chen et al., 2019; Drebes et al., 2016).

The biological relevance of some detected variants remains unclear. An INDEL identified in *G436_RS18190* (Figure 4C) alters the N-terminal sequence and length of a FecR family protein (WP_029757821.1), which may be involved in citrate-mediated iron import (Passmore et al., 2020). A premature stop codon in the gene *G436_RS22205* (Figure 3A) leads to the absence of protein WP_002080799.1 in non-Hardjo serovars. This protein, annotated as a mobile element protein (PF07600 domain), was predicted to be involved in two-component signal transduction and kinase-related activity, potentially contributing to adaptive responses in *L. interrogans* sv. Hardjo. Two-component systems are broadly recognised for their role in sensing environmental signals and modulating stress responses in bacteria (Mitrophanov and Groisman, 2008). Additionally, we identified two INDELs (Figure 4A,B) and one SNP (Figure 3B) differing between groups and leading to the loss of protein-coding sequences in Li-nH group. Affected genes encode hypothetical proteins; nevertheless, due to the lack of identifiable domains or homologs, their potential function remains unclear. Although the biological significance of these variants remains uncertain, their presence in Hardjo and absence in other serovars may reflect differences in regulatory pathways that support host or niche adaptation.

In addition to the high-impact variants, we identified several mutations in pseudogenes (Supplementary Table S3) and missense variants in genes encoding outer membrane proteins (OMPs) (Supplementary Table S4). Pseudogenization is often associated with reduced selective pressure during niche adaptation and has been observed across various host-specialised pathogens (Feng et al., 2022; Pink et al., 2011; Yang et al., 2024). In serovar Hardjo, this process may reflect genome streamlining associated with persistence in ruminant hosts. Missense mutations in putative OMPs, in turn - particularly lipoproteins, which are believed to play key roles in infection and immune evasion - may significantly influence host-pathogen interactions. Even single amino acid change may affect protein function, stability, or surface exposure, warranting further investigation into its potential role in the pathogenicity of *L. interrogans* serovar Hardjo. Therefore, both categories may contribute to further host adaptation, although through different mechanisms.

To complement the SNP and INDEL analyses, we examined orthogroups unique to *L. interrogans* serovar Hardjo, identifying 88 genes not found in other serovars (Figure 5). Functional classification (Figure 6) highlighted differences in categories linked to membrane biogenesis, which is expected, as all genes assigned to this category were located within the *rfb* locus, involved in the synthesis of O-antigen of lipopolysaccharides (LPS) (Cosate et al., 2019). Given the known serovar-specific nature of the *rfb* locus, we focused our analysis on genes located outside this region, where we identified 59 LiH-specific proteins (Table 4). Interestingly, many of the unique LiH-specific genes were found to be clustered in close proximity to the each other, suggesting potential acquisition through horizontal gene transfer (HGT). To explore this hypothesis, we analysed the genomes of serovar Hardjo using IslandViewer 4 to identify putative genomic islands. Despite the presence of mobile element proteins, toxin-antitoxin system components, and a GC content notably higher than the genomic average, the first region could not be classified as a genomic island by IslandViewer 4, and thus no direct evidence of horizontal gene transfer was identified. In contrast, the second region - encompassing multiple hypothetical proteins, an IS1595 transposase, and members of the Sce protein family - was predicted to be a genomic island (GI). This supports the possibility that at least part of the unique genetic content in serovar Hardjo may have been acquired through horizontal gene transfer. Genome plasticity has been shown to contribute to environment and host adaptation in *Leptospira* (Giraud-Gatineau et al., 2024; Moreno et al., 2017).

Although the majority of the LiH-specific orthogroups (37 out of 59 located outside the *rfb* locus) encoded hypothetical proteins, partial functional annotation was possible for a subset of the remaining genes. Among these, four proteins were linked to the toxin-antitoxin (TA) system (WP_000044296.1, WP_000014356.1, WP_002188765.1, WP_000881563.1). TA system seems to play a crucial role in cellular survival under stress conditions. These systems have been linked to various infection-related processes, including toxin production, immune system evasion, and metabolic adaptation, underscoring their potential contributions to pathogenicity (Lopes et al., 2014). Although TA modules are associated with the stabilization of GIs (Munshi et al., 2025), none of the identified TA system components in our dataset were located within predicted genomic islands.

Additionally, one of the LiH-specific orthogroups included an adenylate/guanylate cyclase catalytic domain protein (WP_000288518.1), classified within the ‘Signal transduction mechanisms’ category. Adenylate and guanylate cyclases are enzymes responsible for synthesizing cyclic nucleotides, key secondary messengers involved in environmental sensing and gene regulation (Baker and Kelly, 2004). In *L. interrogans*, cAMP plays a critical function in the regulation of gene expression, particularly during the initial phases of host infection (Matsunaga et al., 2007), whereas c-di-GMP regulates biofilm formation (Thibeaux et al., 2020b). The presence of this gene in the Hardjo-specific set may support its enhanced biofilm-forming capacity, as observed in our *in vitro* assays.

Furthermore, the gene encoding a LiH-specific leucine-rich repeat (LRR) domain-containing protein (WP_002188813.1) was located adjacent to *fliG*. Given the role of FliG in motility control, this genomic arrangement raises the possibility that the LRR-domain protein may influence motility of *L. interrogans* sv. Hardjo, as LRRs have been described as exhibiting a broad spectrum of ligands and having a putative role in bacterial pathogenesis (Foltran et al., 2024).

This study has several limitations that should be acknowledged. First, the analysis was based on a limited number of isolates, which may not fully capture the genomic diversity within the *L. interrogans* sv. Hardjo. Second, while genomic data provide valuable insights into potential phenotypic traits, such predictions remain inherently uncertain without experimental validation. In particular, functional inferences based on bioinformatic tools should be interpreted with caution, especially for genes annotated as hypothetical. Building on these findings, the next phase of our research will involve transcriptomic and proteomic analyses in an animal infection model. These investigations could help identify key genes and their products essential for infection, which could ultimately contribute to the development of more effective control strategies for these infections.

## 5 Conclusion

Our study revealed that *L. interrogans* serovar Hardjo forms a distinct phylogenetic lineage, characterised by specific high-impact mutations and unique gene content. These included a full-length *cas3* gene and a start codon substitution in *thiM*, both of which may contribute to functional differences in Hardjo strains. The Hardjo serovar harbours 88 unique orthologs, most of which are located outside the *rfb* locus. A subset of these cluster within predicted genomic islands, while others are dispersed throughout the genome. These orthologs include genes associated with mobile genetic elements, toxin-antitoxin systems, and signal transduction pathways. Collectively, these genomic features may contribute to the genomic plasticity and adaptive potential of Hardjo strains. Notably, Hardjo strains exhibit significantly enhanced biofilm formation compared to other *L. interrogans* serovars, suggesting a possible genetic basis for improved colonisation of host reproductive tissues.

## Data Availability

The genome assemblies were deposited in GenBank (NCBI) under the following BioProject numbers: PRJNA809530 for strains KR84 (assembly: GCA_022436545.1) and KR85 (assembly: GCA_022436605.1); PRJNA828004 for strain KR40 (assembly: GCA_023158895.3); and PRJNA828002 for strain N116 (assembly: GCA_023515975.1). Further inquiries can be directed to the corresponding authors.
