# Supplementary material for: Serovar-specific genomic features of Leptospira interrogans Hardjo: implications for host adaptation
Source: Front Mol Biosci. 2025 Sep 10;12:1648097. doi: 10.3389/fmolb.2025.1648097 (PMC12457778; doi:10.3389/fmolb.2025.1648097)
Supplement: Supplementary file 1 [file Table1.docx]

| **Serovar of *L.interrogans*** | **Strain** | **Species of origin** | **Country of origin** | **Year of collection** | **Genome status** | **Size (bp)** | **Number of genes** | **GC%** | **RefSeq Assembly Accesion** |
| --- | --- | --- | --- | --- | --- | --- | --- | --- | --- |
| Bataviae | 1489 | Human | Malaysia | 2014 | Complete | 5,349,767 | 4,536 | 35.14 | GCF_014858865.1 |
| Bratislava | PigK151 | Pig | USA | 1989 | Complete | 4,721,584 | 3,875 | 35.03 | GCF_001010765.1 |
| Canicola | 611 | Human | China | 1950 | Complete | 4,755,342 | 3,894 | 35.02 | GCF_008831465.1 |
| Canicola | 782 | Human | Malaysia | 2014 | Complete | 5,308,174 | 4,492 | 35.20 | GCF_014858915.1 |
| Canicola | LJ178 | Dog | China | 2004 | Complete | 4,758,488 | 3,886 | 35.02 | GCF_008831445.1 |
| Copenhageni | FDAARGOS_203 | Human | Brazil | 1996 | Compelete | 4,630,763 | 3,776 | 35.05 | GCF_002073495.2 |
| Copenhageni | Fiocruz L1-130 | Human | Brazil | 1996 | Compelete | 4,627,366 | 3,762 | 35.00 | GCF_000007685.1 |
| Copenhageni | SK-1 | Dog | Saint Kitts and Nevis | 2017 | Compelete | 4,630,180 | 3,778 | 35.04 | GCF_010978155.1 |
| Hardjo | L53 | Cow | Brazil | 2016 | Complete | 4,742,398 | 4,886 | 35.03 | GCF_008118365.1 |
| **Hardjo** | **KR40** | **Horse** | **Italy** | **1997** | **Complete** | **4,793,656** | **3,995** | **35.06** | **GCF_023158895.1** |
| **Hardjo** | **KR84** | **Wallaby** | **United Kingdom** | **1983** | **WGS** | **4,620,945** | **3,888** | **34.97** | **GCF_022436545.1** |
| **Hardjo** | **KR85** | **Dog** | **United Kingdom** | **1982** | **WGS** | **4,627,456** | **3,904** | **34.96** | **GCF_022436605.1** |
| Hardjo | Hardjoprajitno | Human | Indonesia | 1970 | Complete | 4,692,322 | 4,640 | 34.78 | GCF_001443305.1 |
| **Hardjo** | **N 116** | **Cow** | **Belgium** | **2016** | **Complete** | **4,847,508** | **4,015** | **35.05** | **GCF_023515975.1** |
| Hardjo | Norma | Cow | Brazil | 2015 | Complete | 4,762,150 | 4,914 | 35.02 | GCF_001293065.1 |
| Hardjo | OV5 | Sheep | Brazil | 2012 | WGS | 4,775,728 | 5,312 | 35.03 | GCF_001995195.1 |
| Icterohaemorrhagiae | 898 | Human | Malysia | 2014 | Compelete | 4,630,592 | 3,779 | 35.04 | GCF_014858815.1 |
| Lai | Langkawi | Human | Netherlands | 2004 | Compelete | 4,885,316 | 4,070 | 35.22 | GCF_014858895.1 |
| Lai | 56601 | Human | China | *n.i.* | Compelete | 4,698,134 | 3,867 | 35.00 | GCF_000092565.1 |
| Lai | IPAV | n.i. | China | *n.i.* | Compelete | 4,708,530 | 3,867 | 35.03 | GCF_000231175.1 |
| Linhai | 56609 | Human | China | *n.i.* | Compelete | 4,915,652 | 4,056 | 34.64 | GCF_000941035.1 |
| Manilae | UP-MMC-NIID HP | Mouse | Japan | 2012 | Compelete | 4,667,354 | 3,820 | 34.99 | GCF_001047655.1 |
| Manilae | UP-MMC-NIID LP | Mouse | Japan | 2012 | Compelete | 4,667,405 | 3,821 | 34.99 | GCF_001047635.1 |
