# Supplementary material for: Serovar-specific genomic features of Leptospira interrogans Hardjo: implications for host adaptation
Source: Front Mol Biosci. 2025 Sep 10;12:1648097. doi: 10.3389/fmolb.2025.1648097 (PMC12457778; doi:10.3389/fmolb.2025.1648097)
Supplement: Supplementary file 2 [file Table2.docx]

| Strain | Illumina reads | Total bases | Read length | Illumina reads coverage | Nanopore reads | Total bases | Mean reads length | Nanopore reads coverage | Coverage all |
| --- | --- | --- | --- | --- | --- | --- | --- | --- | --- |
| N116 | 3,506,745 | 263,005,875 | 75 | 54.3 | 64,039 | 293,274,234 | 4,437 | 60.5 | 114.8 |
| KR40 | 2,964,691 | 222,351,825 | 75 | 46.4 | 146,849 | 633,546,414 | 4,314 | 132.2 | 178.6 |
| KR84 | 2,674,934 | 200,620,050 | 75 | 41.8 | - | - | - | - | 41.8 |
| KR85 | 2,655,853 | 199,188,975 | 75 | 41.5 | - | - | - | - | 41.5 |
