# Supplementary material for: Serovar-specific genomic features of Leptospira interrogans Hardjo: implications for host adaptation
Source: Front Mol Biosci. 2025 Sep 10;12:1648097. doi: 10.3389/fmolb.2025.1648097 (PMC12457778; doi:10.3389/fmolb.2025.1648097)
Supplement: Supplementary file 3 [file Table5.docx]

| **Protein id** | **Protein name** | **Subcell. Loc.** | **SecP/ SpLip** |  |
| --- | --- | --- | --- | --- |
| **Inside *rfb* locus** | | | | |
| WP_000446476.1 | GNAT family protein | Cytoplasmic |  |  |
| WP_002189147.1 | Putative sugar O-methyltransferase | Cytoplasmic |  |  |
| WP_000900194.1 | Putative sugar O-methyltransferase | Cytoplasmic |  |  |
| WP_025177745.1 | Fkbm family methyltransferase | Cytoplasmic |  |  |
| WP_000041233.1 | Degt/dnrj/eryc1/strs family aminotransferase | Cytoplasmic |  |  |
| WP_000093380.1 | DUF4910 domain-containing protein | Cytoplasmic |  |  |
| WP_000580576.1 | Wbqc family protein | Cytoplasmic |  |  |
| WP_000614938.1 | ATP-binding cassette domain-containing protein | Inner Membrane |  |  |
| WP_000786842.1 | Class I SAM-dependent methyltransferase | Cytoplasmic |  |  |
| WP_000808306.1 | Polysaccharide chain length determinant N-terminal domain-containing protein | Inner Membrane | SpLip+ |  |
| WP_001047905.1 | SIS domain-containing protein | Cytoplasmic |  |  |
| WP_001117632.1 | Degt/dnrj/eryc1/strs family aminotransferase | Cytoplasmic |  |  |
| WP_002076509.1 | Glycosyltransferase family A protein | Cytoplasmic |  |  |
| WP_002078509.1 | N-acetyl sugar amidotransferase | Cytoplasmic |  |  |
| WP_002078520.1 | Degt/dnrj/eryc1/strs family aminotransferase | Cytoplasmic |  |  |
| WP_002098087.1 | Glycosyltransferase 28-like protein | Cytoplasmic |  |  |
| WP_002098115.1 | Gfo/Idh/moca family oxidoreductase | Cytoplasmic |  |  |
| WP_002188948.1 | Aldo/keto reductase // NADP-dependent oxidoreductase domain-containing protein | Cytoplasmic |  |  |
| WP_002188997.1 | SDR family oxidoreductase | Outer Membrane |  |  |
| WP_002189075.1 | O-antigen polymerase | Inner Membrane |  |  |
| WP_002189144.1 | Carbamoyltransferase C-terminal domain-containing protein | Cytoplasmic |  |  |
| WP_002189149.1 | Glycosyltransferase family 4 protein | Cytoplasmic |  |  |
| WP_025177743.1 | Zinc-binding alcohol dehydrogenase | Cytoplasmic |  |  |
| WP_025177744.1 | Hypothetical protein | Cytoplasmic |  |  |
| WP_025177747.1 | Aglz/hisf2 family acetamidino modification protein | Cytoplasmic |  |  |
| WP_025177748.1 | 2OG-Fe(II) oxygenase | Cytoplasmic |  |  |
| WP_002189177.1 | Hypothetical protein | Cytoplasmic |  |  |
| WP_000287382.1 | Class I SAM-dependent methyltransferase | Cytoplasmic |  |  |
| WP_000490616.1 | Hypothetical protein | Cytoplasmic |  |  |
