# Supplementary material for: Serovar-specific genomic features of Leptospira interrogans Hardjo: implications for host adaptation
Source: Front Mol Biosci. 2025 Sep 10;12:1648097. doi: 10.3389/fmolb.2025.1648097 (PMC12457778; doi:10.3389/fmolb.2025.1648097)
Supplement: Supplementary file 4 [file Table3.docx]

| **Locus tag of pseudogene** | **Amino-acid similar sequence (RefSeq)** | **Product of similar amino-acid sequence** | **Identified effect type of variant** |
| --- | --- | --- | --- |
| *G436_RS00635* | WP_010678945.1 | Hypothetical protein | Stop-loss |
| *G436_RS20210* | WP_001010166.1 | Hypothetical protein | Complex (frameshift and stop loss) |
|  |  |  | Stop-loss |
|  |  |  | Complex (frameshift and stop-loss) |
| *G436_RS02575* | WP_002073017.1 | Hypothetical protein | Stop-loss |
| *G436_RS21000* | WP_017854428.1 | SBBP repeat-containing protein | Stop-loss |
| *G436_RS13170* | WP_002102666.1 | Biopolymer transporter tolr | Stop-loss |
| *G436_RS23225* | WP_002108132.1 | DNA-3-methyladenine glycosylase I | Stop-loss |
| *G436_RS11320* | WP_001277634.1 | Hypothetical protein | Stop-gain / Synonymous mutation |
|  |  |  | Frameshift |
| *G436_RS24375* | WP_002102409.1 | Rhomboid family intramembrane serine protease | Stop-gain |
|  |  |  | Frameshift |
| *G436_RS16100* | WP_004771217.1 | Tonb-dependent receptor | Complex (frameshift and stop-gain) / Frameshift |
|  |  |  | Complex (frameshift and stop-gain) / Frameshift |
| *G436_RS24785* | WP_004754593.1 | Hypothetical protein | Complex (frameshift and stop-gain and missense mutation) / Missense mutation |
|  |  |  | Complex (frameshift and stop-gain and missense mutation) / Missense mutation |
| *G436_RS22830* | WP_002124520.1 | IS110 family transposase | Frameshift |
| *G436_RS06955* | WP_017851017.1 | L23 family metallopeptidase | Frameshift |
| *G436_RS12585* | WP_004426262.1 | Hypothetical protein | Frameshift |
| *G436_RS17720* | WP_002070368.1 | DKNYY domain-containing protein | Frameshift |
| *G436_RS19290* | WP_020766257.1 | ATP-dependent 6-phosphofructokinase | Frameshift |
| *G436_RS19880* | WP_004764452.1 | Alpha/beta fold hydrolase | Complex (frameshift and synonymous mutation) / Frameshift |
| *G436_RS24205* | WP_020784391.1 | Hypothetical protein | Stop-loss |
| *G436_RS23920* | WP_002176612.1 | Zinc-binding dehydrogenase | Frameshift |
|  |  |  | Frameshift |
| *G436_RS24645* | WP_002146918.1 | Hypothetical protein | Frameshift |
| *G436_RS23110* | WP_002073879.1 | Hypothetical protein | Frameshift |
| *G436_RS24635* | WP_001975359.1 | Hypothetical protein | Stop-loss |
| *G436_RS07035* | WP_001278774.1 | Hypothetical protein | Stop-loss |
| *G436_RS24730* | WP_002124309.1 | Ankyrin repeat domain-containing protein | Stop-gain |
| *G436_RS17775* | None | NADP-dependent isocitrate dehydrogenase | Complex (frameshift and stop-loss) |
| *G436_RS22615* | WP_000717958.1 | Lipoprotein adhesin ligb | Stop-loss |
| *G436_RS08225* | WP_017857996.1 | Aldo/keto reductase | Stop-loss |
| *G436_RS00495* | WP_020765195.1 | Hypothetical protein | Frameshift |
| *G436_RS20185* | WP_001973331.1 | Hypothetical protein | Complex (frameshift and missense mutation) / Frameshift |
| *G436_RS02515* | WP_000584738.1 | Hypothetical protein | Frameshift |
| *G436_RS21430* | WP_004750281.1 | Type VI secretion system tube protein Hcp | Complex (frameshift and missense mutation) / Frameshift |
| *G436_RS24725* | WP_001072190.1 | SDR family oxidoreductase | Complex (frameshift and missense mutation) / Frameshift |
| *G436_RS22475* | WP_000466210.1 | Hypothetical protein | Frameshift |
| *G436_RS17650* | WP_001222433.1 | Hypothetical protein | Frameshift |
| *G436_RS22805* | WP_017854473.1 | Leucine-rich repeat domain-containing protein | Stop-loss |
| *G436_RS02785* | WP_017856679.1 | Transposase | Complex (frameshift and stop-loss) |
| *G436_RS02975* | WP_035718769.1 | Helix-turn-helix domain-containing protein | Frameshift / Missense mutation |
| *G436_RS24455* | WP_000600948.1 | Hypothetical protein | Frameshift |
| *G436_RS14660* | WP_000550733.1 | Hypothetical protein | Stop-loss |
| *G436_RS24140* | WP_017858525.1 | Acetyltransferase | Stop-loss |
|  |  |  | Stop-loss |
| *G436_RS24385* | WP_000486781.1 | Hypothetical protein | Stop-loss / Missense mutation |
|  |  |  | Stop-loss |
| *G436_RS15610* | WP_017851438.1 | AAA family atpase | Stop-gain |
| *G436_RS23775* | WP_000680352.1 | Leucine-rich repeat domain-containing protein | Stop-gain / Missense mutation / Synonymous mutation |
| *G436_RS16905* | WP_000445690.1 | IR110 family transposase | Stop-gain / Missense mutation |
| *G436_RS24690* | WP_002108899.1 | Transposase | Complex (frameshift and missense mutation) / Frameshift |
| *G436_RS24765* | WP_001141261.1 | Hypothetical protein | Frameshift |
