# Supplementary material for: Serovar-specific genomic features of Leptospira interrogans Hardjo: implications for host adaptation
Source: Front Mol Biosci. 2025 Sep 10;12:1648097. doi: 10.3389/fmolb.2025.1648097 (PMC12457778; doi:10.3389/fmolb.2025.1648097)
Supplement: Supplementary file 5 [file Table4.docx]

| **Type** | **Number of proteins** | | **Protein id** |  |
| --- | --- | --- | --- | --- |
| **SpLip Classification** | | | |  |
| Probable lipoprotein | 56 | | WP_002189382.1, WP_000620483.1, WP_060589200.1, WP_002189272.1, WP_001245092.1, WP_112120680.1, WP_020380898.1, WP_002188180.1, WP_002188449.1, WP_001068429.1, WP_000827839.1, WP_002189024.1, WP_002188957.1, WP_002188355.1, WP_025177659.1, WP_002187969.1, WP_000853292.1, WP_002188684.1, WP_002188692.1, WP_002188730.1, WP_060589304.1, WP_002187589.1, WP_060589322.1, WP_002187552.1, WP_002188452.1, WP_000465286.1, WP_002109295.1, WP_002180959.1, WP_000694948.1, WP_000808723.1, WP_000878637.1, WP_000850108.1, WP_000196640.1, WP_001071889.1, WP_000135128.1, WP_060684633.1, WP_060684553.1, WP_002126405.1, WP_000811111.1, WP_000475802.1, WP_000488365.1, WP_001052452.1, WP_002095068.1, WP_002102726.1, WP_025177713.1, WP_002187685.1, WP_002113022.1, WP_001137994.1, WP_002098079.1, WP_001274115.1, WP_000031889.1, WP_002189379.1, WP_000876503.1, WP_000810425.1, WP_033108701.1, WP_002188074.1 |  |
| Possible lipoprotein | 12 | | WP_060684548.1, WP_002187622.1, WP_002189582.1, WP_025177482.1, WP_000790778.1, WP_000769731.1, WP_002189309.1, WP_001095359.1, WP_217700083.1, WP_000231420.1, WP_161938542.1, WP_000499950.1 |  |
| **SIGNAL P Classification** | | | | |
| LIPO | 66 | | WP_000620483.1, WP_060589200.1, WP_001257938.1, WP_001049440.1, WP_112120680.1, WP_002188180.1, WP_002188449.1, WP_000827839.1, WP_002189020.1, WP_002188355.1, WP_000739010.1, WP_025177659.1, WP_000822424.1, WP_002187969.1, WP_000853292.1, WP_002188081.1, WP_002188692.1, WP_002188730.1, WP_060589304.1, WP_002187589.1, WP_060589322.1, WP_002187552.1, WP_002188452.1, WP_002109295.1, WP_002188951.1, WP_002180959.1, WP_000694948.1, WP_002189037.1, WP_000878637.1, WP_002068529.1, WP_000790778.1, WP_000850108.1, WP_000196640.1, WP_001071889.1, WP_000173801.1, WP_060684633.1, WP_060684553.1, WP_002189309.1, WP_000811111.1, WP_000475802.1, WP_000594559.1, WP_000488365.1, WP_001052452.1, WP_002187771.1, WP_002187863.1, WP_002095068.1, WP_002102726.1, WP_025177713.1, WP_002187685.1, WP_002113022.1, WP_217700083.1, WP_002108174.1, WP_000557286.1, WP_002109016.1, WP_000231420.1, WP_001274115.1, WP_000844347.1, WP_002189379.1, WP_161938542.1, WP_002188372.1, WP_000810425.1, WP_001238409.1, WP_000483732.1, WP_002095755.1, WP_002188074.1, WP_029758863.1 |  |
| SP | 119 | | WP_002189382.1, WP_000885539.1, WP_002189272.1, WP_002073388.1, WP_001245092.1, WP_002189258.1, WP_060684548.1, WP_002189215.1, WP_020380898.1, WP_025177699.1, WP_001068429.1, WP_002188945.1, WP_002189170.1, WP_002188962.1, WP_060589229.1, WP_000478881.1, WP_025177740.1, WP_002188957.1, WP_002188812.1, WP_060684586.1, WP_002188768.1, WP_001070615.1, WP_000487359.1, WP_002188220.1, WP_002187474.1, WP_002187787.1, WP_002187849.1, WP_000875140.1, WP_002187937.1, WP_002187900.1, WP_002187881.1, WP_002187819.1, WP_002188287.1, WP_000836527.1, WP_060684618.1, WP_002105413.1, WP_002189487.1, WP_002188303.1, WP_002188001.1, WP_002121622.1, WP_000746198.1, WP_000858301.1, WP_002162152.1, WP_002187656.1, WP_002187611.1, WP_002187622.1, WP_002188049.1, WP_000465286.1, WP_002188188.1, WP_000915022.1, WP_002188281.1, WP_025177482.1, WP_000808723.1, WP_060589199.1, WP_002189278.1, WP_002188151.1, WP_060684566.1, WP_082283164.1, WP_000846575.1, WP_000466322.1, WP_060684582.1, WP_001080752.1, WP_002187779.1, WP_002188547.1, WP_060684620.1, WP_060684621.1, WP_002188184.1, WP_000135128.1, WP_000873339.1, WP_002189521.1, WP_000532394.1, WP_025177644.1, WP_002187457.1, WP_002187291.1, WP_000769731.1, WP_002188291.1, WP_001247310.1, WP_000885552.1, WP_002126405.1, WP_001095359.1, WP_000720585.1, WP_025177627.1, WP_000286973.1, WP_002188369.1, WP_025177655.1, WP_002188083.1, WP_001188887.1, WP_001188711.1, WP_001083100.1, WP_001049251.1, WP_002187294.1, WP_002189465.1, WP_000282990.1, WP_025177636.1, WP_002187300.1, WP_000847798.1, WP_001258977.1, WP_002098079.1, WP_000717401.1, WP_000017091.1, WP_000809925.1, WP_000691480.1, WP_000682886.1, WP_001232723.1, WP_002070200.1, WP_002081200.1, WP_001075122.1, WP_000093907.1, WP_000949352.1, WP_002092646.1, WP_000876503.1, WP_001220792.1, WP_001024046.1, WP_000281179.1, WP_000499950.1, WP_000744493.1, WP_033108701.1, WP_002187913.1, WP_082280687.1 |  |
| TAT | 4 | | WP_000061047.1, WP_000931513.1, WP_001054402.1, WP_002188008.1 |  |
| TATLIPO | 1 | | WP_001170923.1 |  |
| PILIN | 1 | | WP_000632227.1 |  |
| **SecretomeP Classification** | | | | |
| Non-classical | | 80 | WP_000359692.1, WP_000409985.1, WP_002189335.1, WP_025177694.1, WP_025177696.1, WP_000088216.1, WP_000494959.1, WP_000747128.1, WP_002188983.1, WP_002189108.1, WP_002189142.1, WP_154214849.1, WP_001047265.1, WP_000863304.1, WP_001083714.1, WP_002187788.1, WP_002187830.1, WP_002187851.1, WP_002187919.1, WP_002188680.1, WP_002188684.1, WP_025177773.1, WP_332829146.1, WP_000132187.1, WP_002187550.1, WP_002187551.1, WP_002187577.1, WP_002187653.1, WP_002189585.1, WP_000845535.1, WP_002095384.1, WP_002095657.1, WP_002130790.1, WP_002187380.1, WP_002188672.1, WP_002189259.1, WP_002189338.1, WP_112120683.1, WP_235471960.1, WP_000160490.1, WP_002080049.1, WP_002130493.1, WP_002188959.1, WP_002189580.1, WP_162490608.1, WP_000064872.1, WP_000091878.1, WP_000353669.1, WP_000604761.1, WP_000891789.1, WP_001026912.1, WP_001173872.1, WP_002080535.1, WP_060589271.1, WP_000835447.1, WP_000851097.1, WP_001027463.1, WP_001047666.1, WP_002188575.1, WP_000031889.1, WP_000246381.1, WP_000503780.1, WP_000842066.1, WP_000853160.1, WP_001074436.1, WP_001971818.1, WP_002095567.1, WP_002188600.1, WP_029758581.1, WP_060684554.1, WP_060684595.1, WP_000053885.1, WP_000282718.1, WP_000453267.1, WP_000517946.1, WP_000796630.1, WP_001080165.1, WP_002070182.1, WP_002075983.1, WP_002188275.1 |  |
| **CELLO, PSORT, SOSUI Classification** | | | | |
| Cytoplasmic | 458 | | WP_002189217.1, WP_000076847.1, WP_000619231.1, WP_002189382.1, WP_000885539.1, WP_001001941.1, WP_025177755.1, WP_002189268.1, WP_002189272.1, WP_000606961.1, WP_002189283.1, WP_000764094.1, WP_000412033.1, WP_002189323.1, WP_002189344.1, WP_002189301.1, WP_060684546.1, WP_002189343.1, WP_002189285.1, WP_002189384.1, WP_000291123.1, WP_025177764.1, WP_000613919.1, WP_060684549.1, WP_002189324.1, WP_060684550.1, WP_002188343.1, WP_033108622.1, WP_060684561.1, WP_000639465.1, WP_002188150.1, WP_002188148.1, WP_025177676.1, WP_002188177.1, WP_002188459.1, WP_002188445.1, WP_000791380.1, WP_000622609.1, WP_000619375.1, WP_002188436.1, WP_002188401.1, WP_002187381.1, WP_060684569.1, WP_002187405.1, WP_001106675.1, WP_025177631.1, WP_002187401.1, WP_000036616.1, WP_002189174.1, WP_000348938.1, WP_002189154.1, WP_002189004.1, WP_002189117.1, WP_002189142.1, WP_002189073.1, WP_002189039.1, WP_002106080.1, WP_002188983.1, WP_002188981.1, WP_002189020.1, WP_002189166.1, WP_002068580.1, WP_002189108.1, WP_002189049.1, WP_000033462.1, WP_000058912.1, WP_001070771.1, WP_002189063.1, WP_001001873.1, WP_002188987.1, WP_002080980.1, WP_000286835.1, WP_002187547.1, WP_000099419.1, WP_002188856.1, WP_000747128.1, WP_001202542.1, WP_001062334.1, WP_000279895.1, WP_002188130.1, WP_002188128.1, WP_001050391.1, WP_000234667.1, WP_002188124.1, WP_001191104.1, WP_060684586.1, WP_002188126.1, WP_002188096.1, WP_002188370.1, WP_002131050.1, WP_002188774.1, WP_002188790.1, WP_002188789.1, WP_000433869.1, WP_002188203.1, WP_175618889.1, WP_002187491.1, WP_000979300.1, WP_001084059.1, WP_002187453.1, WP_002187471.1, WP_002187495.1, WP_002187456.1, WP_000582259.1, WP_002187504.1, WP_000819758.1, WP_000418445.1, WP_002187782.1, WP_002187752.1, WP_000205135.1, WP_001190723.1, WP_002187789.1, WP_000568850.1, WP_002187841.1, WP_000653727.1, WP_025177658.1, WP_002187837.1, WP_000438388.1, WP_000875140.1, WP_000716668.1, WP_000174863.1, WP_002187961.1, WP_002187885.1, WP_002188514.1, WP_002188742.1, WP_002188562.1, WP_002188573.1, WP_002188622.1, WP_000503837.1, WP_000028760.1, WP_000456121.1, WP_000998840.1, WP_002188613.1, WP_002188630.1, WP_002188549.1, WP_002188587.1, WP_025177703.1, WP_002189497.1, WP_002189459.1, WP_000470009.1, WP_060684624.1, WP_000954146.1, WP_033108698.1, WP_001083526.1, WP_060589294.1, WP_002189440.1, WP_000681097.1, WP_000280398.1, WP_002188070.1, WP_002188003.1, WP_002188046.1, WP_002188043.1, WP_000067294.1, WP_002188001.1, WP_002130515.1, WP_002188680.1, WP_002188716.1, WP_000581852.1, WP_002188692.1, WP_308340381.1, WP_002187336.1, WP_002187307.1, WP_000155193.1, WP_000047034.1, WP_000253690.1, WP_002187317.1, WP_000863304.1, WP_000892876.1, WP_002189552.1, WP_000095807.1, WP_002189550.1, WP_060589327.1, WP_002189531.1, WP_000684134.1, WP_000052387.1, WP_002187647.1, WP_002187588.1, WP_002187550.1, WP_002187649.1, WP_001222518.1, WP_002187557.1, WP_002187642.1, WP_002187609.1, WP_082280716.1, WP_025177641.1, WP_002187644.1, WP_002187540.1, WP_000201441.1, WP_002108878.1, WP_000132187.1, WP_025177706.1, WP_000026695.1, WP_002188834.1, WP_002188755.1, WP_002188605.1, WP_002188607.1, WP_060589209.1, WP_025177417.1, WP_025177698.1, WP_002189545.1, WP_002189347.1, WP_001012066.1, WP_000848241.1, WP_002188971.1, WP_000694948.1, WP_002188672.1, WP_000654338.1, WP_002095657.1, WP_002189264.1, WP_002079613.1, WP_002126818.1, WP_000278250.1, WP_000576299.1, WP_001148871.1, WP_000807224.1, WP_002189295.1, WP_002189338.1, WP_000578519.1, WP_001199927.1, WP_002189240.1, WP_000744002.1, WP_002188338.1, WP_000802921.1, WP_002109405.1, WP_060684565.1, WP_002187399.1, WP_000845535.1, WP_001013417.1, WP_000510774.1, WP_002189155.1, WP_001161018.1, WP_000118750.1, WP_002189086.1, WP_000450799.1, WP_000779365.1, WP_000466322.1, WP_000394240.1, WP_000230389.1, WP_000021696.1, WP_000615641.1, WP_002188119.1, WP_000007222.1, WP_002188356.1, WP_025177686.1, WP_000058097.1, WP_002188783.1, WP_001113031.1, WP_000780085.1, WP_000702815.1, WP_002080702.1, WP_002079898.1, WP_000623976.1, WP_001192262.1, WP_002187959.1, WP_002130493.1, WP_001011575.1, WP_002108592.1, WP_000639746.1, WP_001071889.1, WP_000450035.1, WP_001098062.1, WP_000512761.1, WP_000873339.1, WP_000121287.1, WP_002187310.1, WP_000929209.1, WP_002080049.1, WP_080035062.1, WP_002187631.1, WP_000820313.1, WP_002187579.1, WP_000169011.1, WP_000391771.1, WP_000111968.1, WP_002187390.1, WP_000050431.1, WP_002188764.1, WP_002188750.1, WP_002188624.1, WP_060684577.1, WP_060684597.1, WP_000601700.1, WP_000371343.1, WP_060684617.1, WP_000639037.1, WP_002188620.1, WP_000130162.1, WP_002079595.1, WP_002065431.1, WP_000555989.1, WP_000098505.1, WP_001228673.1, WP_000064872.1, WP_000453664.1, WP_000010050.1, WP_000832754.1, WP_000280349.1, WP_000720585.1, WP_000990798.1, WP_001008707.1, WP_000286973.1, WP_000458636.1, WP_000517125.1, WP_000653302.1, WP_000841150.1, WP_000566819.1, WP_001027891.1, WP_000353669.1, WP_000559147.1, WP_223813961.1, WP_002188823.1, WP_000654985.1, WP_000365892.1, WP_000808031.1, WP_000893414.1, WP_001084702.1, WP_002188210.1, WP_000488365.1, WP_002187490.1, WP_001011674.1, WP_000483796.1, WP_002187771.1, WP_001097880.1, WP_025177654.1, WP_000538796.1, WP_000683624.1, WP_239044628.1, WP_002095068.1, WP_029759196.1, WP_001170569.1, WP_002188604.1, WP_001153018.1, WP_001273883.1, WP_000594733.1, WP_002188083.1, WP_000805069.1, WP_002163532.1, WP_000572019.1, WP_000943795.1, WP_000930522.1, WP_001052495.1, WP_002069773.1, WP_025177640.1, WP_001031002.1, WP_000777856.1, WP_025177636.1, WP_060684644.1, WP_000098750.1, WP_001027463.1, WP_000238899.1, WP_001081430.1, WP_000466468.1, WP_000554572.1, WP_000600562.1, WP_000575863.1, WP_000054459.1, WP_001088897.1, WP_001026804.1, WP_000836894.1, WP_000741908.1, WP_000202680.1, WP_002189026.1, WP_000239366.1, WP_001258977.1, WP_025177739.1, WP_000831398.1, WP_000196723.1, WP_001289444.1, WP_001970514.1, WP_000895908.1, WP_000927922.1, WP_000877554.1, WP_000231420.1, WP_000212323.1, WP_000599743.1, WP_001146730.1, WP_000728126.1, WP_002187963.1, WP_175618897.1, WP_000944890.1, WP_002188600.1, WP_000126725.1, WP_001977383.1, WP_000274330.1, WP_025177701.1, WP_002189488.1, WP_000098501.1, WP_001109092.1, WP_000371769.1, WP_000506978.1, WP_002101305.1, WP_000619589.1, WP_001971818.1, WP_001070553.1, WP_002188477.1, WP_000729197.1, WP_000136083.1, WP_000837678.1, WP_000853160.1, WP_025177730.1, WP_235471962.1, WP_000695821.1, WP_002189237.1, WP_000589227.1, WP_002189303.1, WP_001257893.1, WP_000090417.1, WP_000612200.1, WP_002081200.1, WP_306795543.1, WP_002105371.1, WP_001033982.1, WP_000375823.1, WP_000890627.1, WP_000212575.1, WP_002187436.1, WP_001178912.1, WP_000376328.1, WP_000402845.1, WP_002188967.1, WP_000949352.1, WP_000683521.1, WP_000993818.1, WP_000655406.1, WP_000136470.1, WP_001089500.1, WP_000855526.1, WP_000447212.1, WP_002187497.1, WP_000347675.1, WP_001115270.1, WP_000502718.1, WP_002187952.1, WP_002188754.1, WP_000841167.1, WP_000149883.1, WP_000406196.1, WP_000762684.1, WP_000627564.1, WP_060589297.1, WP_000013299.1, WP_000243860.1, WP_000423511.1, WP_001080165.1, WP_000776809.1, WP_000119461.1, WP_000234630.1, WP_002070182.1, WP_002189533.1, WP_000682103.1, WP_002187542.1, WP_001181592.1, WP_002074720.1, WP_000905909.1, WP_025177745.1, WP_000549504.1, WP_029758863.1, WP_025177660.1 |  |
| Extracellular | 29 | | WP_001049440.1, WP_002073388.1, WP_020380898.1, WP_025177694.1, WP_002130277.1, WP_002187819.1, WP_002187815.1, WP_002188081.1, WP_002188730.1, WP_060589322.1, WP_002189585.1, WP_002189467.1, WP_002188281.1, WP_060684566.1, WP_112120683.1, WP_000790778.1, WP_000196640.1, WP_002187291.1, WP_002189580.1, WP_000173801.1, WP_000811111.1, WP_025177627.1, WP_000475802.1, WP_002188575.1, WP_002189460.1, WP_025177713.1, WP_002187685.1, WP_002188779.1, WP_002188074.1 |  |
| Outer membraner | 49 | | WP_060684548.1, WP_002189297.1, WP_060684573.1, WP_002189119.1, WP_002188998.1, WP_000478881.1, WP_002189128.1, WP_002128721.1, WP_001047265.1, WP_002187788.1, WP_000739010.1, WP_002187900.1, WP_000777592.1, WP_000836527.1, WP_002188691.1, WP_002188722.1, WP_000746198.1, WP_060589304.1, WP_002187303.1, WP_001083714.1, WP_002162152.1, WP_002187622.1, WP_002187653.1, WP_002188049.1, WP_002109295.1, WP_000915022.1, WP_002187380.1, WP_060684582.1, WP_000480924.1, WP_002188547.1, WP_002188184.1, WP_000135128.1, WP_002109372.1, WP_060684633.1, WP_001247310.1, WP_060589271.1, WP_002188369.1, WP_002188373.1, WP_002080535.1, WP_001052452.1, WP_002189465.1, WP_217700083.1, WP_002189145.1, WP_000017091.1, WP_002095567.1, WP_002070200.1, WP_002188372.1, WP_000499950.1, WP_000744493.1 |  |
| Periplasmic | 12 | | WP_002188180.1, WP_000827839.1, WP_000088216.1, WP_025177773.1, WP_002189582.1, WP_000878637.1, WP_025177644.1, WP_000769731.1, WP_000931513.1, WP_000594559.1, WP_000586168.1, WP_000682886.1 |  |
| Inner Membrane | 130 | | WP_000620483.1, WP_002189357.1, WP_000087372.1, WP_002189260.1, WP_002189327.1, WP_002189365.1, WP_001010479.1, WP_000410888.1, WP_000615573.1, WP_002189581.1, WP_002188449.1, WP_002188461.1, WP_002188422.1, WP_025177629.1, WP_000817966.1, WP_002188964.1, WP_025177735.1, WP_002189022.1, WP_000939573.1, WP_000097922.1, WP_060589243.1, WP_000494959.1, WP_230656329.1, WP_002188115.1, WP_002188355.1, WP_002188371.1, WP_001029466.1, WP_002187494.1, WP_002187508.1, WP_002187487.1, WP_002187477.1, WP_002187793.1, WP_002119423.1, WP_025177659.1, WP_002187937.1, WP_002187978.1, WP_002187910.1, WP_025177729.1, WP_060684618.1, WP_002105413.1, WP_002188535.1, WP_000669545.1, WP_002189443.1, WP_000853292.1, WP_002188067.1, WP_002188068.1, WP_060684631.1, WP_002121622.1, WP_002188010.1, WP_000379430.1, WP_002187311.1, WP_000364367.1, WP_000612019.1, WP_002187552.1, WP_000255840.1, WP_002187408.1, WP_025177775.1, WP_002187313.1, WP_002188329.1, WP_000407109.1, WP_002188840.1, WP_002189278.1, WP_000797207.1, WP_002188441.1, WP_000809845.1, WP_060684568.1, WP_001083855.1, WP_000145878.1, WP_000916822.1, WP_002118504.1, WP_000827960.1, WP_002188825.1, WP_001080347.1, WP_001070492.1, WP_000889674.1, WP_000142583.1, WP_000118558.1, WP_002188539.1, WP_001177672.1, WP_223813960.1, WP_002189521.1, WP_000532394.1, WP_025177504.1, WP_002189400.1, WP_000091878.1, WP_000433097.1, WP_002188818.1, WP_002188127.1, WP_025180781.1, WP_002105649.1, WP_002187843.1, WP_001170202.1, WP_002187858.1, WP_000604090.1, WP_001110375.1, WP_002188310.1, WP_011172390.1, WP_000621287.1, WP_000625160.1, WP_000599987.1, WP_000426718.1, WP_000529868.1, WP_000172205.1, WP_002071771.1, WP_002187403.1, WP_002188943.1, WP_000394959.1, WP_000031029.1, WP_001161189.1, WP_001024276.1, WP_000691480.1, WP_000744956.1, WP_002188603.1, WP_002188545.1, WP_001009543.1, WP_000004844.1, WP_001011704.1, WP_001067450.1, WP_000651421.1, WP_000642916.1, WP_000281179.1, WP_000010045.1, WP_000345339.1, WP_000431254.1, WP_000631578.1, WP_000553166.1, WP_000632686.1, WP_000448519.1, WP_002187582.1, WP_002188275.1 |  |
| Membrane | 56 | | WP_060589200.1, WP_002189215.1, WP_060684560.1, WP_025177699.1, WP_002188453.1, WP_000749830.1, WP_002189006.1, WP_000489550.1, WP_000487359.1, WP_002188220.1, WP_002187474.1, WP_002187787.1, WP_000672053.1, WP_002187849.1, WP_002187881.1, WP_002187830.1, WP_002187969.1, WP_002188171.1, WP_002189519.1, WP_002187646.1, WP_002187656.1, WP_000877309.1, WP_002187611.1, WP_002187589.1, WP_000465286.1, WP_025177482.1, WP_001231962.1, WP_000846575.1, WP_001051000.1, WP_002187779.1, WP_002187872.1, WP_000850108.1, WP_060684620.1, WP_060684621.1, WP_000464907.1, WP_002097744.1, WP_002188959.1, WP_000885552.1, WP_002189309.1, WP_000488887.1, WP_000466091.1, WP_002113022.1, WP_002080901.1, WP_000808183.1, WP_001028159.1, WP_001232723.1, WP_001075122.1, WP_000093907.1, WP_001232577.1, WP_000628890.1, WP_001238409.1, WP_000202857.1, WP_000483732.1, WP_000836888.1, WP_000053885.1, WP_000205854.1 |  |
