# Supplementary figures and images for: Serovar-specific genomic features of Leptospira interrogans Hardjo: implications for host adaptation
Source: Front Mol Biosci. 2025 Sep 10;12:1648097. doi: 10.3389/fmolb.2025.1648097 (PMC12457778; doi:10.3389/fmolb.2025.1648097)

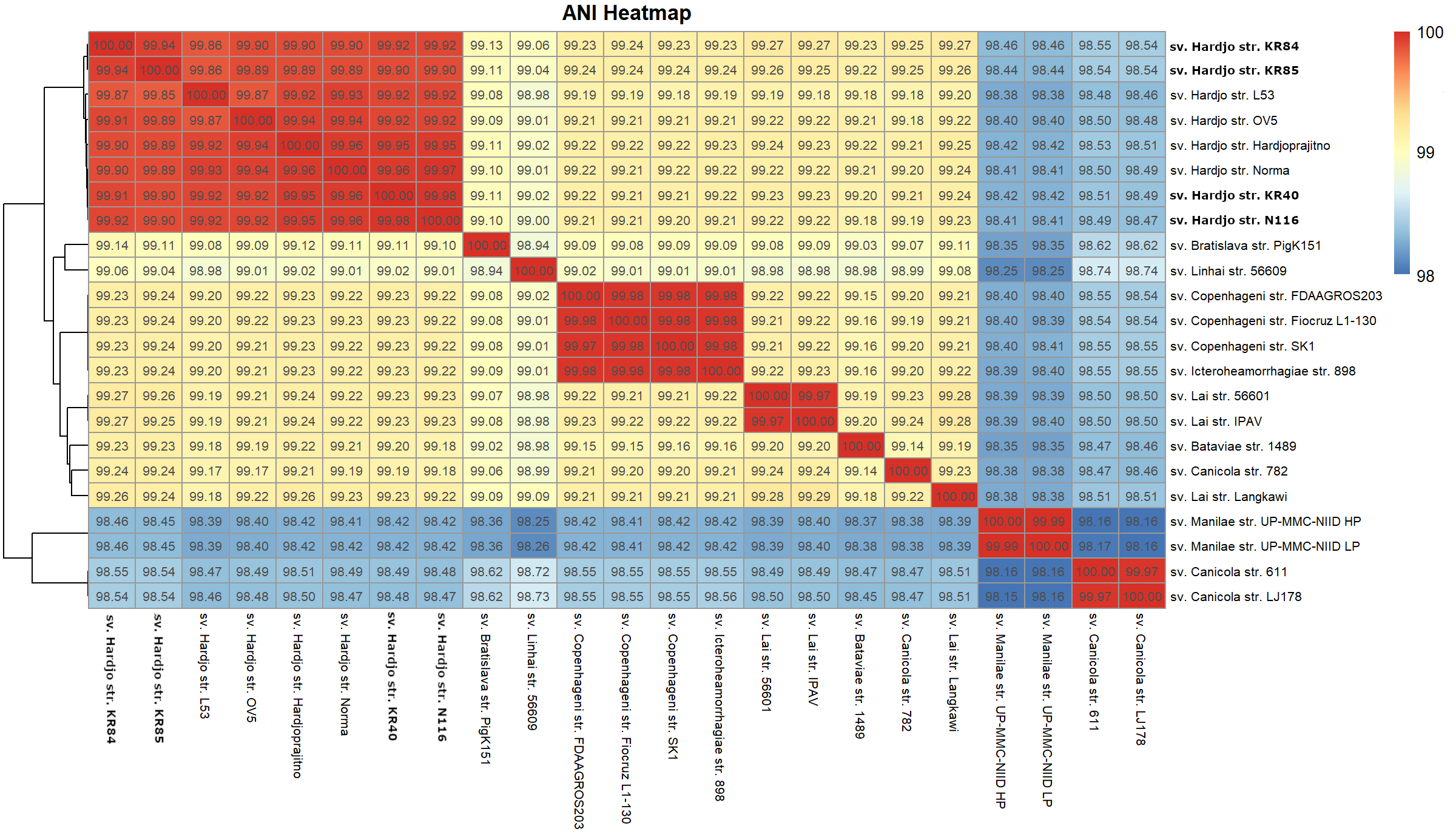

Supplement: Supplementary file 6 [file Image1.png]
